# Comparative analysis of hapalindole, ambiguine and welwitindolinone gene clusters and reconstitution of indole-isonitrile biosynthesis from cyanobacteria

**DOI:** 10.1186/s12866-014-0213-7

**Published:** 2014-08-01

**Authors:** Melinda L Micallef, Deepti Sharma, Brittney M Bunn, Lena Gerwick, Rajesh Viswanathan, Michelle C Moffitt

**Affiliations:** 1 School of Science and Health, University of Western Sydney, School of Science and Health, Locked Bag 1797, Penrith, 2751, NSW, Australia; 2 Department of Chemistry, Case Western Reserve University, 2740 Millis Science Center, Adelbert Road, Cleveland, 44106, OH, USA; 3 Center for Marine Biotechnology and Biomedicine, Scripps Institution of Oceanography, University of California San Diego, La Jolla, 92093, CA, USA

## Abstract

**Background:**

The hapalindole-type family of natural products is a group of hybrid isoprenoid-indole alkaloids, produced solely by members of the Subsection V cyanobacterial strains*.* This family broadly includes the hapalindoles, welwitindolinones, fisherindoles and ambiguines amongst others, all of which have an isonitrile- or isothiocyanate-containing indole alkaloid skeleton, with a cyclized isoprene unit. The hapalindoles are diversified into the welwitindolinones, fischerindoles and ambiguines through the employment of tailoring oxygenase, methyltransferase and prenyltransferase enzymes. We compare the genetic basis for the biosynthesis of this diverse group of natural products and identify key early biosynthetic intermediates.

**Results:**

Whole genome sequencing of freshwater and terrestrial cyanobacteria *Westiella intricata* UH strain HT-29-1, *Hapalosiphon welwitschii* UH strain IC-52-3, *Fischerella ambigua* UTEX 1903 and *Fischerella* sp. ATCC 43239 led to the identification of a candidate hapalindole-type gene cluster in each strain. These were compared with the recently published ambiguine and welwitindolinone gene clusters and four unpublished clusters identified within publicly available genomes. We present detailed comparative bioinformatic analysis of the gene clusters and the biosynthesis of a pivotal indole-isonitrile intermediate resulting in both *cis* and *trans* geometrical isomers. Enzyme analyses and metabolite extractions from two hapalindole-producing *Fischerella* strains indicate the presence of *cis* and *trans* indole-isonitriles as biosynthetic intermediates in the early steps of the pathway.

**Conclusions:**

Interestingly, the organization of the welwitindolinone gene cluster is conserved in all producing strains but distinct from the hapalindole and ambiguine clusters. Enzymatic assays using WelI1 and WelI3 from *Westiella intricata* UH strain HT-29-1 demonstrated the ability to catalyze the formation of both *cis* and *trans* geometrical isomers when using a cell lysate. The enzymatic and metabolic characterization of both *cis* and *trans* indole-isonitrile intermediates implies conservation of their stereochemical integrity towards members of the ambiguine and welwitindolinone products. In summary, we present data that supports a unified biosynthetic pathway towards hapalindoles in nine individual species of cyanobacteria. Diversification of the pathway occurs later through the employment of specialized enzymatic steps towards fischerindoles, ambiguines and welwitindolinones.

## Background

The hapalindole family of natural products is a group of hybrid isoprenoid-indole alkaloids. Specifically, the hapalindole family has been identified solely within the genera *Hapalosiphon, Fischerella, Westiella* and *Westiellopsis*[1], which belong to the Subsection V (also known as Stigonematales) order of cyanobacteria*.* The hapalindole-type natural products are a structurally fascinating group of compounds, with over 80 variations identified to date, and is defined by the presence of an isonitrile- or isothiocyanate-containing indole alkaloid skeleton, with a cyclized isoprene unit. Members of the hapaldinole family are then divided into several sub-families, which include the hapalindoles, welwitindolinones, fisherindoles, ambiguines, fischambiguines, hapalindolinones, hapaloxindoles and fontonamides [1]*.*

**Figure 1:**
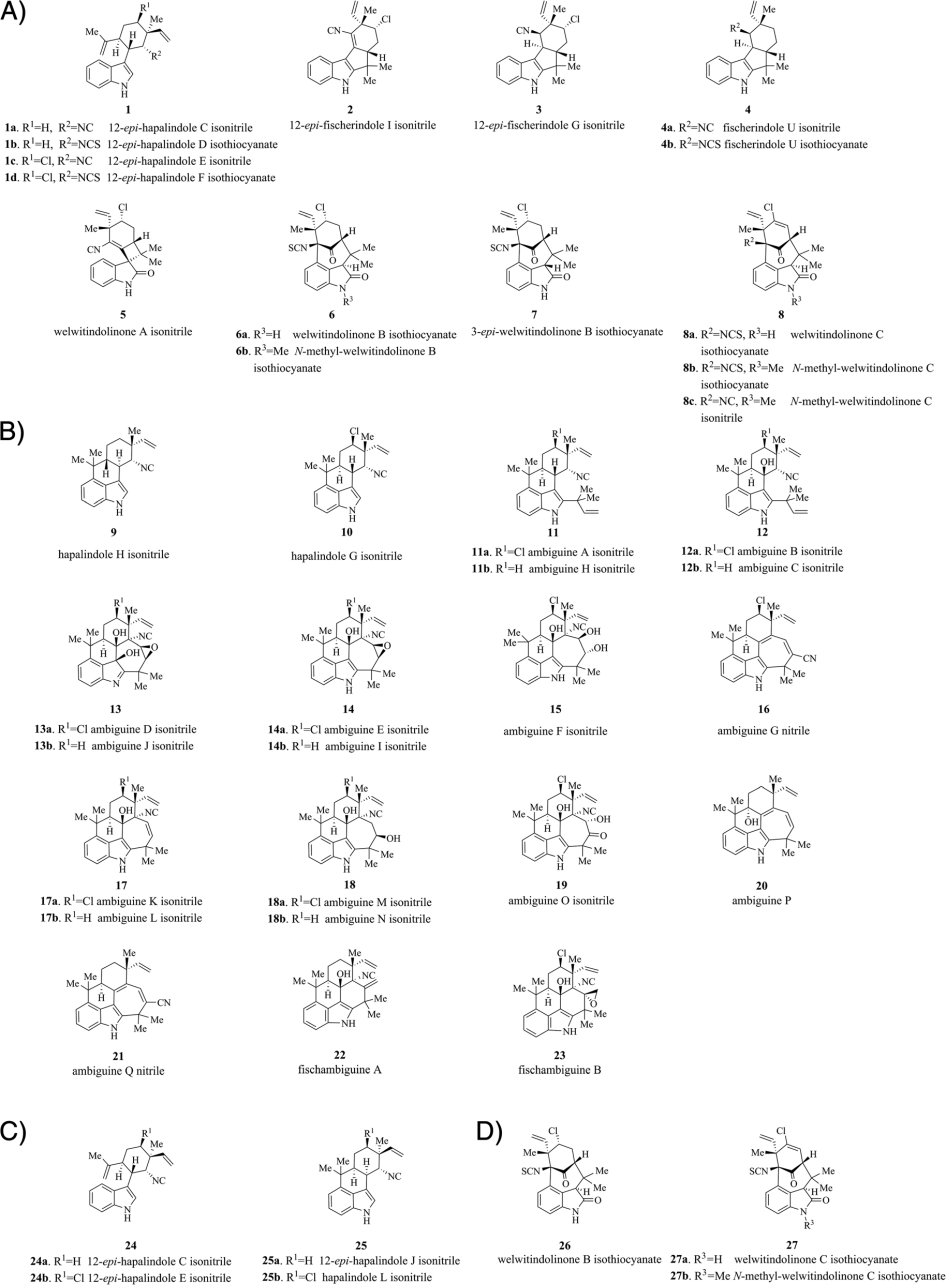
**Structures of hapalindole family of natural products isolated from the strains sequenced in this study. A)** Hapalindoles, fischerindoles and welwitindolinones isolated from *Hapalosiphon welwitschii* UH strain IC-52-3. **B)** Hapalindoles, ambiguines and fischambiguines isolated from *Fischerella ambigua* UTEX 1903. **C)** Hapalindoles isolated from *Fischerella* sp. ATCC 43239. **D)** Welwitindolinones isolated from *Westiella intricata* UH strain HT-29-1.

Structural diversity within the hapalindole family is generated through variation in the pattern of terpene cyclization, chlorination, methylation, oxidation/reduction and additional prenylation. Remarkably, despite their structural similarities, each analogue displays unique bioactivities, ranging from anticancer bioactivity by *N*-methyl welwitindolinone C isothiocyanate (Figure 1, 8b/27b) [2],[3], to antituberculosis activity of ambiguines K and M, fischambiguine B (Figure 1, 17a, 18a, 23) [4],[5] and hapalindoles X and A [6].

Recently, gene clusters responsible for ambiguine (*amb*) and welwitindolinone (*wel*) biosynthesis were identified from *Fischerella ambigua* UTEX 1903 and *Hapalosiphon welwitschii* UTEX B1830, respectively [7],[8]. Key biosynthetic steps towards the formation of hapalindoles were characterized. *In vitro* characterization of AmbP3 confirmed the *amb* gene cluster was responsible for the biosynthesis of the ambiguines from hapalindole G [7]. Furthermore, *in vitro* characterization of a methyltransferase, WelM, encoded only within the *wel* gene cluster, confirmed its involvement in the methylation of welwitindolinone C isothiocyanate to form *N*-methylwelwitindolinone C isothiocyanate [8].

In order to further investigate the relatively complex network of biosynthetic pathways leading to the biosynthesis of the hapalindole-type natural products, we chose to analyze four Subsection V cyanobacterial strains known to produce a range of these compounds (Figure 1). *Fischerella sp.* ATCC 43239 has been reported to produce four hapalindoles [9], whereas *Fischerella ambigua* UTEX1903 produces a range of hapalindoles, ambiguines and fischambiguines [4],[5]. Multiple hapalindoles, fischerindoles and welwitindolinones have been reported to be produced by *Hapalosiphon welwitschii* UH strain IC-52-3, whilst three welwitindolinones have been reported from *Westiella intricata* UH strain HT-29-1 [10] (Figure 1). We aimed to identify a gene cluster responsible for the biosynthesis of these compounds in each strain, while also screening publicly available cyanobacterial genomes for the presence of the hapalindole-type biosynthetic gene cluster. The genetic analyses were complemented by *in vitro* enzymatic assays for the isonitrile biosynthesis enzymes WelI1 and WelI3, resulting in the formation of both *cis* and *trans* isoforms of 3-(2-isocyanovinyl)indole (hereafter known as indole-isonitrile). Furthermore, the enzymology is supported through structural verification of both *cis* and *trans* isoforms of the indole-isonitrile extracted directly from *Fischerella* sp. and *Fischerella ambigua* cultures.

## Results and discussion

Whole genome sequencing of *Fischerella* sp. ATCC 43239 (hereafter known as FS ATCC43239), *Fischerella ambigua* UTEX 1903 (hereafter known as FA UTEX1903), *Hapalosiphon welwitschii* UH strain IC-52-3 (hereafter known as HW IC-52-3) and *Westiella intricata* UH strain HT-29-1 (hereafter known as WI HT-29-1) was used to identify a gene cluster encoding the biosynthesis of the hapalindoles (precursor molecules for fischerindole, ambiguine and welwitindolinone biosynthesis) in each strain. Candidate gene clusters were identified in all four sequenced genomes, and PCR reactions were used to seal any gaps. The *wel (wel*witindolinone) gene cluster was identified in the genome of WI HT-29-1 (Additional file 1: Table S1), and in the genome of HW IC-52-3 (Additional file 1: Table S2). The *hpi* (*h*a*p*al*i*ndole) gene cluster was identified in the FS ATCC43239 genome (Additional file 1: Table S3).

The ambiguine (*amb*) gene cluster was recently published by Hillwig *et al.*[7]. We independently sequenced and identified the *amb* gene cluster from the genome of FA UTEX1903 as part of this study. While the majority of the nucleotide sequence is 100% identical, some differences upstream of the 3’ end of *ambE3* were identified. The *amb* gene cluster from Hillwig *et al*. [7] encodes ParA and ParB family chromosome partitioning proteins and transposases, however, the *amb* gene cluster sequenced in this study does not contain these genes, instead, genes encoding monooxygenases and oxidoreductases were identified (Additional file 1: Table S4).

There are currently 11 Subsection V cyanobacteria draft genomes that are publicly available. We screened all Subsection V genomes in an attempt to identify any additional gene clusters encoding the biosynthesis of the hapalindole group of compounds. There has been no reported investigation of hapalindole-type natural products from these strains. We have identified a gene cluster from *Fischerella* sp. PCC 9339 (hereafter known as FS PCC9339) (Additional file 1: Table S5), *Fischerella* sp. PCC 9431 (hereafter known as FS PCC9431) (Additional file 1: Table S6) and *Fischerella muscicola* SAG 1427-1 (hereafter known as FM SAG1427-1) (Additional file 1: Table S7) (Table 1).

**Table 1:** **Comparison of the nine** ***hpi*,** ***amb*** **and** ***wel*** **biosynthetic gene clusters**

**Name of organism**	**Length of gene cluster (kb):**	**Number of genes:**	**Name of gene cluster:**	**Reference:**
*Fischerella sp.* ATCC 43239	40.2	30	*hpi*	This study
*Fischerella sp.* PCC 9339	44.9	35	*hpi*	This study
*Fischerella ambigua* UTEX 1903	42	32	*amb*	[7]
*Fischerella ambigua* UTEX 1903	50.7	37	*amb*	This study
*Hapalosiphon welwitschii* UTEX B1830	36	30	*wel*	[8]
*Westiella intricata* UH strain HT-29-1	59.3	47	*wel*	This study
*Hapalosiphon welwitschii* UH strain IC-52-3	55.8	45	*wel*	This study
*Fischerella sp.* PCC 9431^*^	57.1	45	*wel*	This study
*Fischerella muscicola* SAG 1427-1	25.1	20	*wel*	This study

*The exact length of this gene cluster was unable to be determined due to sequencing gaps in two genes located at the 5’ end of the gene cluster.

**Figure 2:**
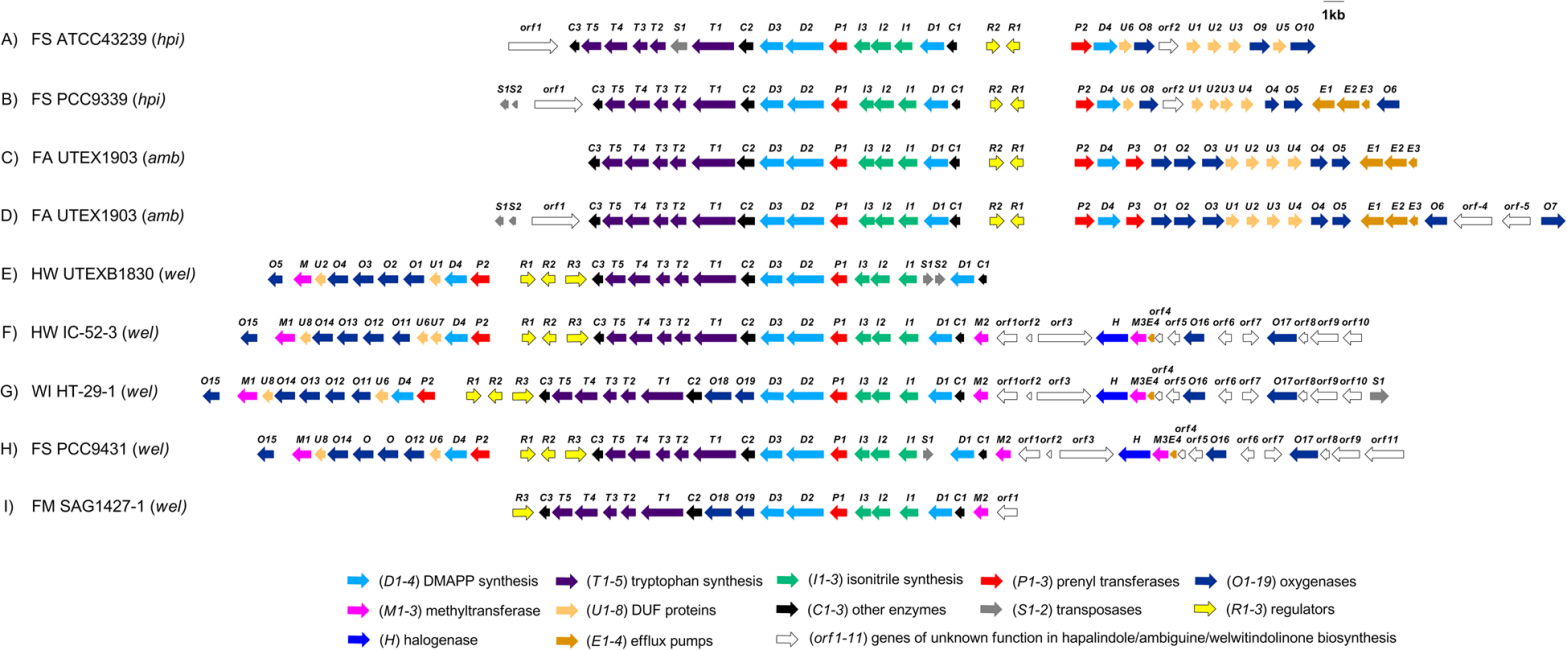
**Illustration of the hapalindole (*hpi*), ambiguine (*amb*) and welwitindolinone (*wel*) biosynthetic gene clusters. A)***hpi* gene cluster from *Fischerella* sp. ATCC 43239 (this study). **B)***hpi* gene cluster from *Fischerella* sp. PCC 9339 (JGI IMG/ER: 2516653082). **C)***amb* gene cluster from *Fischerella ambigua* UTEX 1903 [7]. **D)***amb* gene cluster from *Fischerella ambigua* UTEX 1903 (this study). **E)***wel* gene cluster from *Hapalosiphon welwitschii* UTEX B1830 [8]. **F)***wel* gene cluster from *Hapalosiphon welwitschii* UH strain IC-52-3 (this study). **G)***wel* gene cluster from *Westiella intricata* UH strain HT-29-1 (this study). **H)***wel* gene cluster from *Fischerella* sp. PCC 9431 (JGI IMG/ER: 2512875027). **I)***wel* gene cluster from *Fischerella muscicola* SAG 1427-1 (JGI IMG/ER: 2548876995).

Prior to submission of this manuscript, the identification and characterization of the *wel* gene cluster from *H. welwitschii* UTEX B1830 was published by Hillwig *et al.*[8] (hereafter known as HW UTEXB1830). As the nucleotide sequence was not available at the time of submission, we were unable to perform any analysis using this data. However, based on the image presented in the manuscript, this gene cluster demonstrates remarkable similarity to the *wel* gene clusters identified in this study (Figure 2).

### Comparisons of the *hpi, amb* and *wel* gene clusters

The identification of these seven gene clusters, along with the recently published *amb* and *wel* gene clusters, allows genetic comparisons to be performed. The nomenclature of genes used in this report follows those in the previously published *amb* and *wel* gene clusters [7],[8]. For simplicity, a gene common to all gene clusters is referred to only by the corresponding letter and number. We have identified a core set of 19 genes common to the cyanobacterial strains analyzed in this study (Table 2). These common genes include the tryptophan biosynthesis genes *T1-5* and *C2*, the isonitrile biosynthesis genes *I1-3*, the isoprenoid biosynthesis genes *D1-4*, the geranyl pyrophosphate synthase gene *P2*, the hapalindole-specific aromatic prenyltransferase gene *P1*, the regulatory protein-encoding genes *R1* and *R2*, as well as *C1* and *C3* (which encode other enzymes). These 19 genes share greater than 92% sequence identity at the protein level.

**Table 2:** Protein names, putative function, and % identity of the encoded Hpi, Amb and Wel enzymes

**Enzyme**	**FS ATCC 43239**	**FS PCC 9339**	**FA UTEX 1903**	**HW IC-52-3**	**WI HT-29-1**	**FS PCC 9431**	**FM SAG 1427-1**	% identity*
**Tryptophan biosynthesis:**								
TrpE	HpiT1	HpiT1	AmbT1	WelT1	WelT1	WelT1	WelT1	93.3
TrpC	HpiT2	HpiT2	AmbT2	WelT2	WelT2	WelT2	WelT2	92
TrpA	HpiT3	HpiT3	AmbT3	WelT3	WelT3	WelT3	WelT3	92.7
TrpB	HpiT4	HpiT4	AmbT4	WelT4	WelT4	WelT4	WelT4	95.7
TrpD	HpiT5	HpiT5	AmbT5	WelT5	WelT5	WelT5	WelT5	94.8
DAHP synthase	HpiC2	HpiC2	AmbC2	WelC2	WelC2	WelC2	WelC2	95.3
**IPP and DMAPP biosynthesis:**								
Dxr	HpiD1	HpiD1	AmbD1	WelD1	WelD1	WelD1	WelD1	96.4
Dxs	HpiD2	HpiD2	AmbD2	WelD2	WelD2	WelD2	WelD2	97.7
IspG	HpiD3	HpiD3	AmbD3	WelD3	WelD3	WelD3	WelD3	98.7
IspH	HpiD4	HpiD4	AmbD4	WelD4	WelD4	WelD4	-	95.3
**Isonitrile biosynthesis:**								
IsnA	HpiI1	HpiI1	AmbI1	WelI1	WelI1	WelI1	WelI1	94
IsnA	HpiI2	HpiI2	AmbI2	WelI2	WelI2	WelI2	WelI2	96.2
IsnB	HpiI3	HpiI3	AmbI3	WelI3	WelI3	WelI3	WelI3	95.6
**Prenyltransferases:**								
Aromatic prenyltransferase	HpiP1	HpiP1	AmbP1	WelP1	WelP1	WelP1	WelP1	96.9
GPP	HpiP2	HpiP2	AmbP2	WelP2	WelP2	WelP2	-	93
Aromatic prenyltransferase	-	-	AmbP3	-	-	-	-	-
**Methyltransferases:**								
N-methyltransferase	-	-	-	WelM1	WelM1	WelM1	-	98.8
SAM-dependent methyltransferase	-	-	-	WelM2	WelM2	WelM2	WelM2	91.2
Histamine N-methyltransferase	-	-	-	WelM3	WelM3	WelM3	-	99
**Regulation proteins**								
Response regulator containing a CheY-like receiver domain and an HTH DNA-binding domain	HpiR1	HpiR1	AmbR1	WelR1	WelR1	WelR1	-	93.4
Transcriptional regulator, LuxR family	HpiR2	HpiR2	AmbR2	WelR2	WelR2	WelR2	-	96.2
Response regulator with CheY-like receiver domain and winged-helix DNA-binding domain	-	-	-	WelR3	WelR3	WelR3	WelR3	93.3
**Other:**								
Dephospho-CoA kinase-like protein	HpiC1	HpiC1	AmbC1	WelC1	WelC1	WelC1	WelC1	93.2
Phosphoglycerate mutase family protein	HpiC3	HpiC3	AmbC3	WelC3	WelC3	WelC3	WelC3	96.4
**Transporter genes:**								
DevC protein	-	HpiE1	AmbE1	-	-	-	-	98.2
ABC exporter membrane fusion protein, DevB family	-	HpiE2	AmbE2	-	-	-	-	99.7
Conserved membrane hypothetical protein	-	HpiE3	AmbE3	-	-	-	-	100
Small multidrug resistance protein	-	-	-	WelE4	WelE4	WelE4	-	97.8

*The % identity is based on comparison of all enzymes sequenced.

### Organization of genes

Comparison of the gene organization of the *hpi/amb/wel* gene clusters identified groups of genes whose order and orientation are conserved, however, the presence/absence of specific genes distinguish the *hpi, amb* and *wel* gene clusters from each other (Figure 2). These differences are probably essential in forming the given hapalindole-type natural products.

Within the *hpi/amb/wel* gene clusters analyzed, there appears to be two major transcripts, which were predicted based on the direction of the genes (Figure 2). The first predicted major transcript begins at *C1.* There are 15 genes (*C1, D1, I1, I2, I3, P1, D2, D3, C2, T1, T2, T3, T4, T5* and *C3*) present on this predicted transcript in all nine gene clusters, in which the arrangement and orientation of the genes has been conserved. However, there are additional genes located within this predicted transcript in a few strains. In the *hpi* gene cluster from FS ATCC43239, there is a single transposase located between *T2* and *T1.* There are two transposases located between *I1* and *D1* in the *wel* gene cluster from HW UTEXB1830, and there is a single transposase located between *I1* and *D1* in the gene cluster from FS PCC9431. There are also two oxygenase genes, *O18* and *O19,* located between *C2* and *D3* in the gene clusters from WI HT-29-1 and FM SAG1427-1. The gene clusters from WI HT-29-1, HW IC-52-3, FS PCC9431 and FM SAG1427-1 also contain two additional conserved genes (*orf 1* and *M2*), located at the beginning of this predicted transcript. In some gene clusters, o*rf2* is also located at the beginning of this predicted transcript. Given that the known welwitindolinone-producing strains contain these genes on the same predicted transcript with several other key genes in the biosynthetic pathway, these additional genes may be important in the biosynthesis of the welwitindolinones.

The second predicted major transcript in the *hpi/amb/wel* gene clusters begins with the gene *P2* and is present in all the gene clusters identified in this study, except the gene cluster from FM SAG1427-1. In the *hpi* and *amb* gene clusters, this major predicted transcript is located upstream of the 5’ end of *C1,* however, in the *wel* gene clusters, the predicted transcript is located downstream of the 3’ end of *C3* (Figure 2)*.* A number of oxygenase genes and sequence-redundant domain of unknown function (DUF) genes are found on these predicted transcripts, which vary between each gene cluster. The differences in these oxygenase and DUF genes are likely related to differences in the natural products produced.

There are additional predicted transcripts in the gene clusters from FS PCC9339 and the *amb* gene clusters. Downstream of the 3’ end of *O5,* the exporter genes *E1, E2,* and *E3* are all potentially transcribed on a single transcript. In the gene cluster from FS PCC9339 and the *amb* gene cluster sequenced in this study, the gene *O6* is also possibly located on this transcript. In the *amb* gene cluster sequenced in this study, *O7* is predicted to be located on a separate transcript.

The genes clusters from HW IC-52-3, WI HT-29-1 and FS PCC9431 contain five additional predicted transcripts upstream of the 5’ end of *orf2,* which are highly conserved (greater than 98% identity at the nucleotide level). These genes are not located in any of the *hpi* or *amb* gene clusters, suggesting these genes may encode proteins responsible for welwitindolinone biosynthesis.

The regulatory genes *R1, R2* and *R3* do not seem to form an operon, and the arrangement and orientation of these genes between each other are conserved in the gene clusters from HW UTEXB1830, HW IC-52-3, WI HT-29-1 and FS PCC9431.

By comparing the identified hapalindole-like natural products with their encoded gene clusters and proposed biosynthesis, the presence/absence of specific genes may be used to predict which class of hapalindole-type natural products (either hapalindole, ambiguines or welwitindolinones) may be produced from newly identified gene clusters. For example, the presence of AmbP3 suggests the ability to produce the ambiguines. This knowledge was used to infer the biosynthesis of the hapalindole-type natural products from FS PCC9339, FS PCC9431 and FM SAG1427-1, since the metabolite profile of these organisms has not been determined. It is likely that the gene cluster from FS PCC9339 encodes the biosynthesis of the hapalindoles, and the gene clusters from FS PCC9431 and FM SAG1427-1 encode the biosynthesis of the welwitindolinones. The gene cluster from FM SAG1427-1 was grouped with the *wel* gene clusters based on the presence and high similarity of the genes *O18, O19, R3* and *M2,* all of which are specific to the *wel* gene clusters. However, the genes located on either side of the *wel* gene cluster from FM SAG1427-1 display no similarity to other genes in the *wel* gene clusters, and some highly conserved genes are missing. The absence of conserved core *wel* genes suggests the gene cluster may be non-functional in this strain.

**Figure 3:**
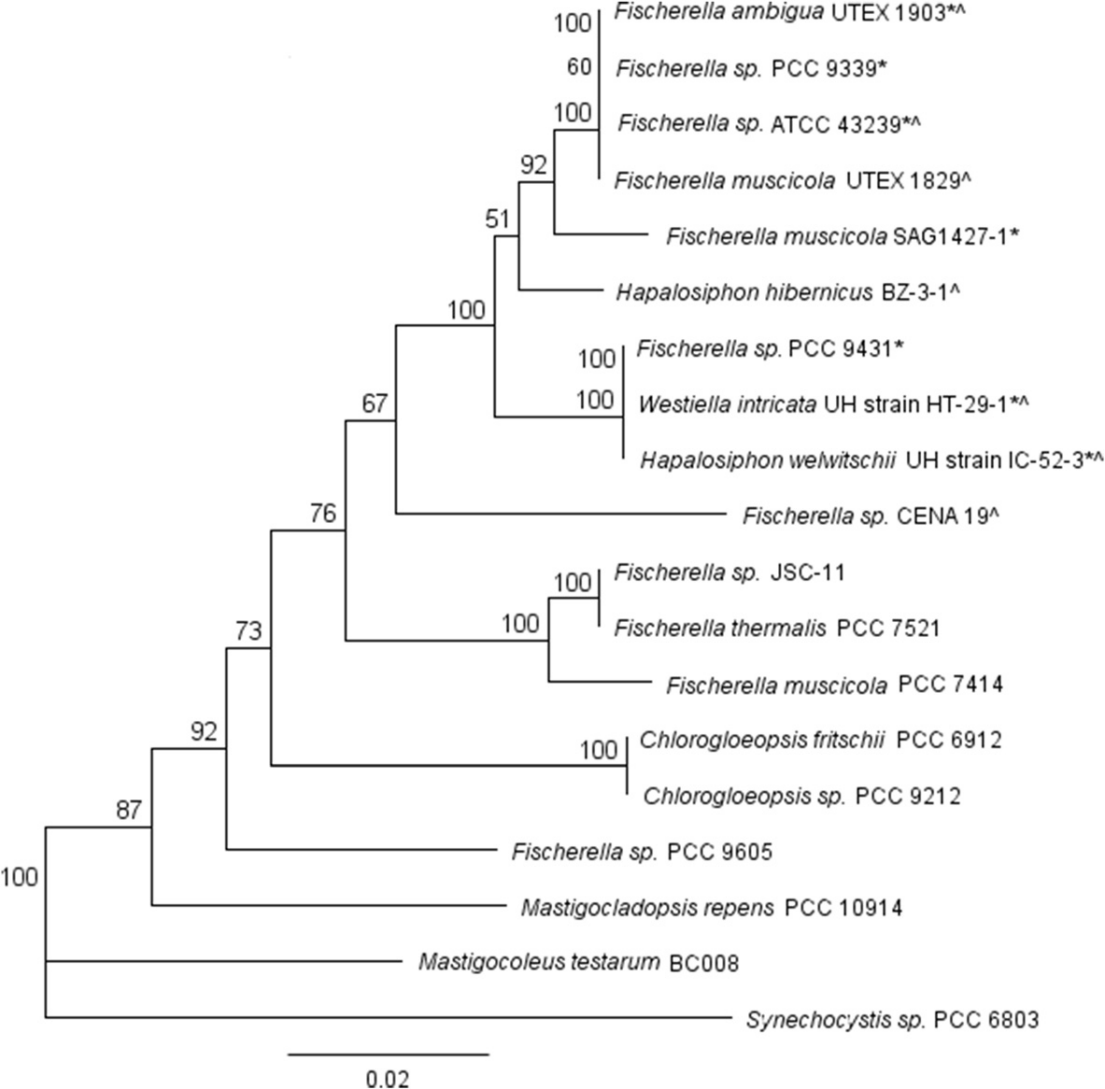
**Phylogenetic analysis of Subsection V strains using 16S rDNA.** Phylogenetic tree was constructed using a 929 bp fragment of the 16S rRNA gene from Subsection V cyanobacterial strains analyzed in this study, Subsection V cyanobacterial strains for which the genome has been sequenced, and cyanobacterial strains known to produce hapalindole-type natural products. *Fischerella muscicola* UTEX 1829 [GenBank: AB075984], *Fischerella* sp. PCC 9339 [IMG Gene ID: 2517062088], *Fischerella* sp. ATCC 43239 [GenBank: KJ768872], *Fischerella ambigua* UTEX 1930 [GenBank: KJ768871], *Fischerella muscicola* SAG 1427-1 [GenBank: AB075985], *Fischerella* sp. PCC 9431 [IMG Gene ID: 2512976007], *Hapalosiphon welwitschii* UH strain IC-52-3 [GenBank: KJ767019], *Westiella intricata* UH strain HT-29-1 [GenBank: KJ767016], *Hapalosiphon hibernicus* BZ-3-1 [GenBank: EU151900], *Fischerella* sp. CENA 19 [GenBank: AY039703], *Fischerella* sp. JSC-11 [GenBank: HM636645], *Fischerella thermalis* PCC 7521 [GenBank: AB075987], *Fischerella muscicola* PCC 7414 [GenBank: AB075986], *Chlorogloeopsis fritschii* PCC 6912 [GenBank: AB093489], *Chlorogloeopsis fritschii* PCC 9212 [GenBank: AB075982], *Fischerella* sp. PCC 9605 [IMG Gene ID: 2516144612], *Mastigocladopsis repens* PCC 10914 [GenBank: AJ544079], *Mastigocoleus testarum* BC 008 [IMG Gene ID: 2264826627] and *Synechocystis* sp. PCC 6803 [GenBank: NR_074311]. *indicates *hpi*/*amb*/*wel* gene cluster was identified in these strains. ^ indicates these strains are known producers of hapalindole-family of natural products. *Synechocystis* sp. PCC 6803 was used as the outgroup. Phylogenetic trees were constructed using the Geneious Tree Builder program, using the neighbour-joining method. Numbers at each branch point are the bootstrap values for percentages of 100 replicate trees.

In order to assess the mechanism of inheritance of *hpi/amb/wel* gene clusters within the Subsection V strains, we performed phylogenetic analysis of the 16S rDNA (Figure 3). All of the strains that either contain the *hpi/amb/wel* gene cluster or are known producers of these molecules appear to be a monophyletic group, indicating that the gene cluster first appeared in a single ancestral strain. This is interesting, considering that some well-studied cyanobacterial natural products, such as microcystin and saxitoxin, exhibit a scattered distribution across several genera [11],[12]. Studies suggest that the scattered distribution of these molecules occurs as a result of horizontal gene transfer [11]–[13]. The hapalindole family of molecules, however, appears to have been only inherited vertically to each of the descendant strains. This pattern of inheritance is also supported by a phylogenetic tree constructed using the prenyltransferase P1 protein sequence, which shows a similar clustering of sequences to the 16S rDNA tree (Additional file 2). The conserved inheritance of these gene clusters implies that the hapalindole family of compounds plays an important role in the producing strains.

### Tryptophan biosynthesis

Five of the six essential genes required for the biosynthesis of L-tryptophan from chorismate, which are paralogues of *trpABCDE* (*T1-5*), were identified in all nine biosynthetic gene clusters [14]. The sixth gene, *trpF,* a phosphoribosylanthranilate isomerase gene, is located outside of the gene cluster consistently in all strains analyzed. Analysis of the genomes sequenced in this study revealed some cyanobacterial strains also contain a second set of genes which encode for tryptophan biosynthesis, however, other strains only contain the tryptophan genes within the gene cluster for tryptophan biosynthesis. Another gene common to all nine gene clusters is *C2,* a DAHP (3-deoxy-D-arabinoheptulosonate-7-phosphate) synthase gene, which encodes an enzyme regulating the biosynthesis of DAHP from the condensation of PEP (phosphoenolpyruvate) and erythrose-4-phosphate, the first enzymatic step of aromatic amino acid synthesis [15].

### Indole-isonitrile biosynthesis

A signature chemical feature of the hapalindole family of alkaloids is the presence of an isonitrile functional group. The specific location of this functional group is conserved across all isonitrile-containing members and is at C11 of the hapalindole core (except for ambiguine G and Q, where it has transposed into a nitrile functionality). The isonitrile biosynthesis genes *I1-3* were identified and found to be tightly conserved in all clusters (greater than 94% identity at the protein level across all gene clusters analyzed in this study). The gene products of *I1* and *I2* demonstrate high sequence similarity to the previously characterized isonitrile synthases, IsnA (from an uncultured organism) [16] and PvcA (from *Pseudomonas aeruginosa* PA01) [17]. The six core motifs of IsnA and PvcA were identified in I1 and I2 (Additional file 3). The gene product of *I3* displayed high sequence similarity to the α-ketoglutarate-dependent oxygenase, IsnB and PvcB [16],[17]. We identified the amino acids of the metal-binding motif in all of the encoded protein sequences of I3 (Additional file 4).

Pathways encoded by Isn and Pvc require only one copy of each gene for the effective production of the isonitrile functional group from tryptophan [16],[17]. However, all strains investigated in this study have a duplicated copy of *I1* (*I2*), with at least 78% identity between them at the protein level*.* Recent characterization of the set of isonitrile biosynthetic enzymes from the *amb* gene cluster identified that the enzymes AmbI1 and AmbI3 are responsible for the biosynthesis of the isonitrile functional group, however, the enzyme AmbI2 is functionally-redundant in isonitrile biosynthesis [7]. It is curious that this arrangement of three genes, containing the duplicated *I1,* has been maintained across all strains with very little evidence of mutation over time.

**Figure 4:**
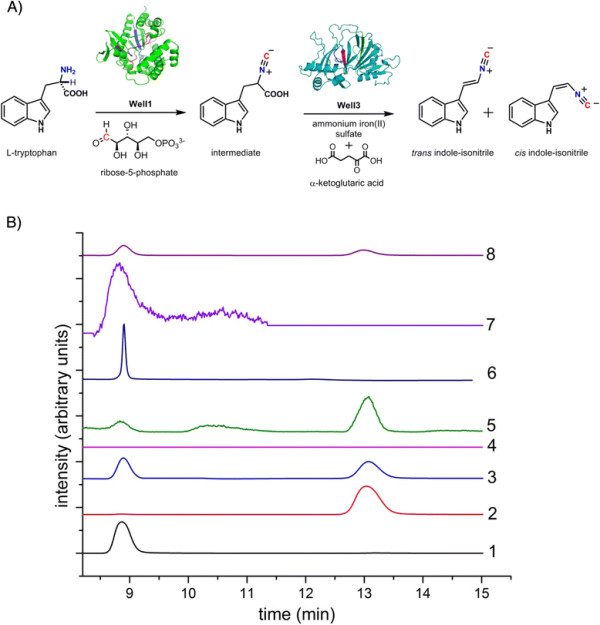
**HPLC analyses of WelI1-WelI3 catalyzed indole-isonitrile formation. A)** Biosynthetic steps catalyzed by WelI1 and WelI3 respectively. *In vitro* reconstitution assay for indole-isonitrile biosynthesis using cell lysates of *E. coli* BL21(DE3) heterologously expressing WelI1 and WelI3. Models of WelI1 and WelI3 were built based on homology to PvcA and PvcB X-ray structures [34] using Phyre2.0. **B)** HPLC was analyzed at 310 nm with a UV detector. X-axis – retention time in minutes (min). Y-axis - intensity in arbitrary units. Presented as a stacked Y-plot and is drawn to relative intensity units. B1) Synthesized *cis* indole-isonitrile only (t_R_ = 8.8 min). B2) Synthesized *trans* indole-isonitrile only (t_R_ = 13.1 min). B3) Co-injection of synthetic standards of *cis* and *trans* indole-isonitrile. B4) Control for enzyme assay where cell lysates of *E. coli* BL21(DE3) were subjected to assay conditions without WelI1 and WelI3. B5) WelI1 and WelI3 enzyme assay after 16 h incubation at 25°C. B6) Control sample (4) spiked with *cis* indole-isonitrile after 3 h incubation. B7) Control sample (4) spiked with *cis* indole-isonitrile after 16 h incubation. B8) Co-injection of *cis* and *trans* indole-isonitrile with enzyme assay mixture. Peaks show only relative intensities and are not normalized for concentration of metabolites.

In order to establish the biosynthetic function of WelI1/I3 from the *wel* gene cluster of WI HT-29-1, these proteins were heterologously expressed and biosynthetic assays were performed using the *Escherichia coli* cell lysates (expressing WelI1/I3) with the proposed substrates L-tryptophan and ribose-5-phosphate, in the presence of ammonium iron sulfate and α-ketoglutaric acid (Figure 4, A) [18]. An assay containing both enzymes was preferred to individual assays based on the instability of the first intermediate (L-Trp-isonitrile) during isolation (Figure 4, A) [18]. Prior to analyzing the enzymatic assay mixtures, chemically synthesized *cis* and *trans* isomers of indole-isonitrile (Additional file 5) were first identified as distinct traces with unique retention times (Figure 4, B1-3). HPLC analyses of enzymatic reaction mixtures after incubation for 16 h showed the presence of two major peaks, confirming the production of the *cis* and *trans* isomers of indole-isonitrile (Figure 4, B5). A non-enzymatic formation of the indole-isonitrile was ruled out based on a negative control (no WelI1/I3) (Figure 4, B4). Synthesized *cis* indole-isonitrile standard was incubated under the assay conditions as controls to test if isomerization was involved. Results indicate that the *trans* isomer is not formed through an *E. coli*-mediated isomerization (Figure 4, B6 and 7). However, there is a significant amount of degradation of the *cis* isomer observed after 16 h of incubation as compared to analysis after 3 h (Figure 4, B7 compared to 6). As a further measure of validation, we co-injected *cis* and *trans* indole-isonitriles to samples where enzymatic product formation was observed (Figure 4, B8) and only the two product peaks that correlated to the retention times of *cis* and *trans* indole-isonitriles were observed. Finally, additional confirmation for indole-isonitrile biosynthesis was obtained through LC-MS analyses under negative ion-mode (Additional file 6). Overall, the assay results validated the formation of *cis* and *trans* indole-isonitriles as the biosynthetic products of the pathway encoded by WelI1/I3. In contrast to AmbI1/3 and WelI1/3 from FA UTEX1903 and HW UTEXB1830 respectively, which only produce the *cis* isomer of the indole-isonitrile [7],[8], assay mixtures containing WelI1/I3 from WI HT-29-1 produced both the *cis* and *trans* isomers of the indole-isonitrile when the assay is carried out over a 16 h duration. Because a mixture of *cis* and *trans* products are observed for the first time, these are exciting observations from a natural product biosynthesis point of view, as they lead to interesting questions about the biochemical mechanism of WelI1/I3. It is probable that the enzymes are producing the *trans* isoform in concentrations below detection limit within the first 3 h, which then accumulate over time and can be detected after 16 h. However, it remains to be seen whether both of these isomers engage as substrates for downstream hapalindole-producing steps of the pathway.

**Figure 5:**
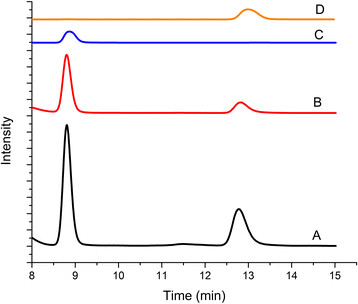
**HPLC analyses of both*cis*and*trans*isomers of indole-isonitrile from culture extracts.** HPLC was analyzed at 310 nm with a UV detector. X-axis – retention times in minutes (min). Y-axis refers to intensity in arbitrary units. Plot presented as a stacked Y-plot and is drawn to relative intensity units. **A)** FS ATCC43239 extracts. **B)** FA UTEX1903 extracts. **C)** Synthesized *cis* isomer (t_R_ = 8.8 min). **D)** Synthesized *trans* isomer (t_R_ = 13.1 min). Peaks show only relative intensities and are not normalized for concentration of metabolites.

Until now, direct evidence of the presence of indole-isonitriles from cyanobacterial cultures has remained elusive. Therefore, we set out to identify the presence of isonitrile intermediates through direct extraction from FS ATCC43239 and FA UTEX1903 cultures. Using synthetic standards, similar to the assay described above, a HPLC method was established in order to verify the presence of indole-isonitriles from cyanobacterial biomass. HPLC analyses identified both the *cis* and *trans* isomers of the indole-isonitrile in the extracts of FS ATCC43239 and FA UTEX1903 (Figure 5). To confirm the HPLC results, FS ATCC43239 and FA UTEX1903 cultures were extracted and analyzed by LC-MS under negative ion mode electrospray ionization, and the organic extract from both cultures displayed a [M-H]^+^ peak at *m/z* 167 consistent with that observed from the chemically synthesized standard. The HRESI-MS characterization for the synthesized indole-isonitrile displayed a parent [M]^+^ peak at *m/z* 168.0689 (expected *m/z* =168.0687), while culture extracts from FS ATCC43239 and FA UTEX1903 displayed an indole-isonitrile [M]^+^ peak at *m/z* 168.0685 (within 5.95×10^-8^ units from synthesized sample = 59 ppb) (Additional file 7). Thus, we report for the first time, the presence of both *cis* and *trans* isomers of indole-isonitrile in two *Fischerella* cultures as biosynthetic intermediates of the hapalindole pathway.

In concurrence with our enzymology observations, the detection of both *cis* and *trans* isomers from cyanobacterial extracts by HPLC analysis raised the possibility of inter-conversions and/or thermal isomerizations during the timescale of the analyses. Therefore, to rule out these possibilities, we subjected the *cis* isomer of the indole-isonitrile from synthesized standard to the identical extraction protocol performed on the native cyanobacterial cells. Only thermal degradation (no isomerization) was observed (similar to the enzymology observation over 16 h). Overall, the stereochemical integrity of the individual *cis* and *trans* isomers was found to be maintained through the course of our isolation procedures.

Hapalindole products isolated from FS ATCC43239 strain display both *cis* and *trans* stereodisposition in their C10-C11 arrangement (Figure 1, 24a-b versus 25a-b), implying that both isomers are probable substrates in the subsequent step of the biosynthetic pathway. The presence of the *trans* isomer in extracts from FA UTEX1903 is intriguing considering ambiguines possess strictly a *cis* stereodisposition between C10-C11 stereocenters.

### Geranyl pyrophosphate biosynthesis

The other proposed substrate for hapalindole biosynthesis is geranyl pyrophosphate (GPP), which is biosynthesized from isopentenyl pyrophosphate (IPP) and dimethylallyl pyrophosphate (DMAPP) *via* the MEP/DOXP pathway. Four genes (*D1-4*) show homology to *dxs, dxr, ispG* and *ispH,* which are proposed to biosynthesize IPP and DMAPP from pyruvate and D-glyceraldehyde-3-phosphate. The remaining genes, *ispDEF,* are located outside of the gene cluster in most strains (*ispE* was not identified in the genomes of FS ATCC43239, FA UTEX1903 and FS PCC9339). IPP and DMAPP are the substrates for the enzyme geranyl pyrophosphate synthase (GPP synthase) to produce GPP [19]. The gene *P2* is also conserved across most gene clusters and was proposed to encode a GPP synthase. Recent enzymatic characterization of AmbP2 and WelP2 from the *amb* and *wel* gene clusters, respectively, confirmed our prediction that P2 encodes a GPP synthase [7],[8].

### Hapalindole-specific prenyltransferase

The *P1* gene is also part of the core set of genes found in each of the *hpi/amb/wel* gene clusters. *P1* encodes a putative prenyltransferase with sequence similarity to other previously characterized proteins belonging to the ABBA superfamily of prenyltransferases [20]. Sequence analysis of P1 revealed the absence of the Mg-dependent prenyl diphosphate binding motif (N/D)DXXD [21]. The prenyltransferase P1 in the *hpi/amb/wel* gene clusters was initially proposed to convert GPP (biosynthesized by P2) to *β*-ocimene in order to catalyze the prenylation of indole-isonitrile to produce 12-*epi*- hapalindole C [10]. Based on the biosynthetic schemes proposed by Moore and others, we anticipated P1 to possess activity that catalyzes a reverse prenylation independent of any additional enzymatic participation, in which C3, rather than C1, of *β*-ocimene is attached to C10 of indole-isonitrile (hapalindole numbering) [1],[10]. Recent characterization of AmbP1 and WelP1 from the *amb* and *wel* gene clusters both failed to convert GPP to *β*-ocimene [7],[8]. We independently set out to characterize P1 from the *wel* gene cluster from WI HT-29-1. WelP1 was incubated with possible substrates tryptophan or indole-isonitriles with GPP at a range of temperatures and incubation times, however, no differences between the control (no enzyme) and assay were detected *via* LC-MS. As no product was detected, we suspected an additional enzyme was probably involved. We proposed that the enzymatic pathway for hapalindole biosynthesis involves P1 for GPP binding and activation, simultaneously coupled with a halogenating enzyme, based upon the presence of a halogenated prenyl group. A putative halogenase (WelH) in the *wel* gene clusters from HW IC-52-3, WI HT-29-1 and FS PCC9431 displays similarity to FADH_2_-dependent halogenases, containing both a FAD-binding motif (GxGxxG) and a tryptophan-binding motif (WxWxIP) [22]. FADH_2_-dependent halogenases require a partner enzyme, a flavin reductase, to regenerate reduced flavin from FAD and NADH [23],[24]. Located upstream from *welH, orf9* is homologous to NADH dehydrogenases, which may be a partner enzyme for WelH. This partner gene set (*welH* and *orf9*) is conserved between WI HT-29-1, HW IC-52-3 and FS PCC9431 with greater than 98% sequence identity at the protein level. Due to the absence of sequence data downstream of the published *wel* gene cluster from HW UTEXB1830 we were unable to establish the presence of a homologous halogenase in this strain [8].

In order to test our theory that WelH was involved in hapalindole biosynthesis, we overexpressed WelH from the *wel* gene cluster from WI HT-29-1. We used SsuE as the flavin reductase, as SsuE is commonly used as a flavin reductase with other FADH_2_-dependent halogenases from diverse genera [24]. However, biochemical assays with WelH and SsuE did not result in a halogenated product. Additionally, biochemical assays using WelP1, WelH and SsuE were also unsuccessful. The absence of this halogenase from the *hpi* and *amb* gene clusters suggests that *welH* may not be involved in hapalindole biosynthesis. Recent reports by Hillwig *et al.*[8] suggest that the oxygenase WelO5 (numbering based on those in Hillwig *et al*. [8], not this paper, see below) might function to perform this role. Further investigation is required to determine the additional enzymes required for hapalindole biosynthesis with P1.

### Oxygenase genes

Comparison of the *hpi, amb* and *wel* gene clusters also identified 37 genes encoding oxygenases from all eight gene clusters (excluding *wel* from HW UTEXB1830). Each encoded protein sequence was compared to each other, and those with an identity greater than 90% were believed to be homologous proteins, and labelled with the same number (Additional file 8). A total of 19 different oxygenase genes (*O1-19*) were identified (Table 3). Eleven of the 19 oxygenases (*O1-4, O8-9, O11-14* and *O19*) were identified as Rieske-type oxygenase genes. The [2Fe-2S] cluster motif, the iron-sulfur Rieske domain and nonheme Fe(II)-binding motif were identified within the encoded protein sequence (Additional file 9). Both HpiO4 and AmbO4 appear to be atypical Rieske-homologous proteins. Analysis of all 19 oxygenase genes revealed none were common in all nine gene clusters. *O1-3* and *O7* were found exclusively in the *amb* gene cluster, suggesting these oxygenases are involved in the structural diversification of the ambiguines. *O4-6* were identified in the *hpi* gene cluster from FS PCC9339 and the *amb* gene cluster. Furthermore, *O8* was found exclusively in both of the *hpi* gene clusters identified in this study. Two oxygenases, *O9* and *O10,* were identified only in the *hpi* gene cluster from FS ATCC43239. *O12* and *O14-17* were identified in three *wel* gene clusters (HW IC-52-3, WI HT-29-1 and PCC9339), and *O11* and *O13* have been identified in the *wel* gene cluster from WI HT-29-1 and HW IC-52-3. The presence of *O11* and *O13* in the *wel* gene cluster from FS PCC9431 was unable to be confirmed due to sequencing gaps in the oxygenase genes located at the 5’ end of the gene cluster. Two oxygenase genes, *O18* and *O19,* proposed to encode a monooxygenase and a Rieske-type oxygenase, were identified in the *wel* gene clusters from WI HT-29-1 and FM SAG1427-1. Further biochemical investigation is required to determine the specific role of each oxygenase to their respective pathway.

**Table 3:** **List of encoded oxygenase enzymes from the** ***hpi*,** ***amb*** **and** ***wel*** **biosynthetic gene clusters**

**Enzyme**	**FS ATCC 43239**	**FS PCC 9339**	**FA UTEX 1903**	**HW IC-52-3**	**WI HT-29-1**	**FS PCC 9431**	**FM SAG 1427-1**	**% identity**
Oxygenases								
Rieske oxygenase	-	-	AmbO1	-	-	-	-	-
Rieske oxygenase	-	-	AmbO2	-	-	-	-	-
Rieske oxygenase	-	-	AmbO3	-	-	-	-	-
Rieske oxygenase	-	HpiO4	AmbO4	-	-	-	-	100
Oxidoreductase, 2OG-Fe(II) oxygenase family	-	HpiO5	AmbO5	-	-	-	-	98.1
Alkanesulfonate monooxygenase	-	HpiO6	AmbO6	-	-	-	-	100
Oxidoreductase, FAD dependent pyridine nucleotide disulfide	-	-	AmbO7	-	-	-	-	-
Rieske oxygenase	HpiO8	HpiO8	-	-	-	-	-	100
Rieske oxygenase	HpiO9	-	-	-	-	-	-	-
Oxidoreductase, FAD dependent	HpiO10	-	-	-	-	-	-	-
Rieske oxygenase	-	-	-	WelO11	WelO11	-	-	90.9
Rieske oxygenase	-	-	-	WelO12	WelO12	WelO12	-	99.1
Rieske oxygenase	-	-	-	WelO13	WelO13	-		97.8
Rieske oxygenase	-	-	-	WelO14	WelO14	WelO14	-	98.1
Oxidoreductase, 2OG-Fe(II) oxygenase family	-	-	-	WelO15	WelO15	WelO15	-	96.3
Indoleamine 2,3-dioxygenase	-	-	-	WelO16	WelO16	WelO16	-	99.0
Choline dehydrogenase-like flavoprotein	-	-	-	WelO17	WelO17	WelO17	-	99.0
Monooxygenase	-	-	-	-	WelO18	-	WelO18	99.0
Rieske oxygenase	-	-	-	-	WelO19	-	WelO19	98.3

### Genes containing a domain of unknown function

Another common feature of the *hpi/amb/wel* gene clusters is the presence of DUF genes. 21 DUF genes were identified from all of the gene clusters (excluding HW UTEXB1830) and each protein sequence was compared to each other and those with an identity greater than 90% were labelled with the same number (Additional file 10). A total of eight different genes (*U1-8*) were identified (Table 4). Although one DUF gene was not found in all gene clusters, *U6* was identified in all of the *hpi* and *wel* gene clusters. *U1-3* were identified in both the *hpi* and *amb* gene clusters, and *U4* was identified in the *hpi* gene cluster from FS PCC9339 and the *amb* gene cluster. *U5* was identified exclusively in the *hpi* gene cluster from FS ATCC43239, *U7* was identified only in the *wel* gene cluster from HW IC-52-3, and *U8* was identified in the *wel* gene clusters from HW IC-52-3, WI HT-29-1 and FS PCC9431. However, as the function of these protein-encoding genes remains unknown, their involvement in the biosynthesis of the hapalindole, fischerindoles, ambiguines and welwitindolinones remains elusive.

**Table 4:** **List of unknown proteins with domain of unknown function from** ***hpi*,** ***amb*** **and** ***wel*** **clusters**

**Enzyme**	**FS ATCC 43239**	**FS PCC 9339**	**FA UTEX 1903**	**HW IC-52-3**	**WI HT-29-1**	**FS PCC 9431**	**FM SAG 1427-1**	**% identity**
Unknown proteins with DUF								
Unknown function	HpiU1	HpiU1	AmbU1	-	-	-	-	97.7
Unknown function	HpiU2	HpiU2	AmbU2	-	-	-	-	98
Unknown function	HpiU3	HpiU3	AmbU3	-	-	-	-	99.1
Unknown function	-	HpiU4	AmbU4	-	-	-	-	100
Unknown function	HpiU5	-	-	-	-	-	-	-
Unknown function	HpiU6	HpiU6	-	WelU6	WelU6	WelU6	-	94.2
Unknown function	-	-	-	WelU7	-	-	-	-
Unknown function	-	-	-	WelU8	WelU8	WelU8	-	97.9

### Methytransferase genes

The *wel* gene clusters identified in WI HT-29-1, HW IC-52-3 and FS PCC9431 contain three genes with homology to different methyltransferases (*welM1*, *welM2* and *welM3*) (Table 2). Only *welM2* was identified in the *wel* gene cluster from FM SAG1427-1. Although sequence downstream of the *wel* cluster in HW UTEXB1830 is unable to establish the presence of *welM2* and *welM3,* we propose (on the basis of the homology of genes within each of the *wel* gene clusters) that *welM2* and *welM3* would be conserved. Hillwig *et al.*[8] have established that *welM1* encodes the *N*-methyltransferase involved in the biosynthesis of *N*-methyl-welwitindolinone C isonitrile via *in vitro* enzymology, confirming the *wel* gene cluster is responsible for welwitindolinone biosynthesis. *M2* is proposed to encode a SAM-dependent methyltransferase, whilst *M3* is proposed to encode a histamine *N*-methyltransferase. The purpose of *welM2* and *welM3* remain unknown, as no other known compounds of the hapalindole family require an additional methylation reaction.

### Ambiguine biosynthesis

The aromatic prenyltransferase AmbP3 was characterized, and shown to be responsible for catalyzing the prenylation of hapalindole G with DMAPP to produce the ambiguines. We identified *ambP3* only in the *amb* gene cluster from FA UTEX1903, thus confirming this is the only species within this study with the capability to produce ambiguines.

### Other genes

Three response regulator-coding genes have been identified from the nine gene clusters analyzed in this study. *welR3* is unique to the *wel* gene clusters. However, the two regulatory genes *R1* and *R2* were identified in all *hpi/amb/wel* gene clusters (excluding FM SAG1427-1). The transporter genes *E1-3* that were originally identified in the *amb* gene cluster have also been identified in the *hpi* gene cluster from FS PCC9339. *E4*, proposed to encode a small multidrug resistance protein, was identified in three *wel* gene clusters identified in this study (HW IC-52-3, WI HT-29-1 and FS PCC9431). *C1* and *C3* are proposed to encode proteins for which their function in hapalindole/ambiguine/welwitindolinone biosynthesis remains unknown.

## Conclusions

The identification of the seven biosynthetic gene clusters in this study, along with the recently published *amb* and *wel* biosynthetic gene clusters, enabled bioinformatic comparisons to be performed. Organization of the *wel* gene clusters is distinct from the *hpi* and *amb* gene clusters, which enables the prediction of which class of hapalindole-type natural products (either hapalindoles, ambiguines or welwitindolinones) may be biosynthesized from these clusters within genomes. Phylogenetic analysis indicates organisms that contain the *hpi/amb/wel* gene clusters form a monophyletic clade and, thus, hapalindole biosynthesis is likely to be inherited vertically, rather than horizontally. Though a conserved triad of genes (*I1*-*I3*) are present in all clusters, WelI1 and WelI3 are sufficient to catalyze the resulting formation of *cis* and *trans* geometrical isomers when using a cell lysate. This first report of the isolation of both *cis* and *trans* geometrical isomers for the indole-isonitrile from both enzymatic assays using WelI1 and I3 from WI HT-29-1 and from metabolic extractions of two hapalindole-producing *Fischerella* strains, implies the conservation of stereochemical integrity towards members of the ambiguine and welwitindolinone products, and opens new mechanistic possibilities to be studied.

This study reports new findings which are essential to the overall elucidation of the unusual mechanism of biosynthesis of the hapalindole family of compounds, however, several steps still remain elusive. At present, only a few group V cyanobacterial genomes are available. However, as more genomes are sequenced from cyanobacteria known to produce hapalindole-type natural products and further enzymology is performed, the full biosynthetic pathway to all the hapalindole-type natural products may be determined. A diverse range of oxygenases have been identified in the gene clusters reported in this study. The future enzymatic characterization of the oxygenases will most likely provide a foundation to elucidate the complex biosynthetic pathway of the hapalindole-type natural products.

## Methods

### Cyanobacterial culturing

The cyanobacterial strains WI HT-29-1 and HW IC-52-3 were obtained from the University of Hawaii cyanobacterial culture collection, FS ATCC43239 from American Type Culture Collection and FA UTEX1903 from Culture Collection of Algae at the University of Texas at Austin. All cyanobacterial cultures were maintained in Blue-Green 11 (BG-11) medium [25] (Fluka, Buch, Switzerland). WI HT-29-1 and HW IC-52-3 cultures were maintained at 24°C with 12 h light/dark cycles illuminated with 11 μmol m^-2^ s^-1^ of photons. FS ATCC43239 and FA UTEX1903 were illuminated with 80-100 μmol m^-2^ s^-1^ of photons on a 18:6 h light/dark cycle at 22°C.

For extraction and isolation of biosynthetic intermediates, cyanobacterial cultures were grown in 18-20 L of BG-11 media and 4% CO_2_ mixed in air was bubbled through the cultures following inoculation.

### Genomic DNA extraction

Prior to genomic DNA (gDNA) extraction, WI HT-29-1 and HW IC-52-3 cyanobacterial cells were first filtered using a 3 μm nitrocellulose membrane (Millipore, North Rhyde, Australia) to remove heterotrophic bacteria and washed with 200 mL of sterile BG-11 media. gDNA was extracted from WI HT-29-1 and HW IC-52-3 cyanobacterial cells following the protocol outlined in Morin *et al.*[26]. RNA was removed using 2 μL of ribonuclease A (≥70 Kunitz U/mg) and incubated at room temperature for 15 min. Phenol:chloroform:isoamyl alcohol extraction was performed and gDNA was precipitated using isopropanol and resuspended in TE buffer. Additional polysaccharides were removed following the protocol outlined in Wilson [27]. FS ATCC43239 gDNA was isolated following the protocol described in Ausubel *et al.*[28] and FA UTEX1903 gDNA was extracted following a protocol described in Mustafa [29].

### Whole genome sequencing and bioinformatics

High molecular weight gDNA from WI HT-29-1 and HW IC-52-3 was sent to BGI (Beijing Genome Institute, China) for genome sequencing *via* high throughput Illumina sequencing technology. BGI performed genome assembly and gene annotation using Glimmer v3.0.

Extracted gDNA from FA UTEX1903 and FS ATCC43239 was submitted to Case Western Reserve Genomics Core Facility for whole genome sequencing. Paired end DNA libraries were obtained by using Nextera DNA sample preparation kit and sequenced using the Illumina GAIIx platform. Raw reads quality was assessed using FastQC 0.10.1 (Babraham Bioinformatics) with default settings and trimmed with Seqyclean 1.3.12 (http://cores.ibest.uidaho.edu/software/seqyclean). Filtered reads were assembled *de novo* using the velvet package (Version 1.2.08) and a kmer range between 55-63. The optimal assembly based on expected genome size, N_50_ and contig number was used for downstream annotation and analysis. Gene annotation was performed by BGI using Glimmer v3.2. A Basic Local Alignment Search Tool (BLAST) search was performed to identify the putative function of proteins based on sequence similarity [30].

Nucleotide and protein sequences were organized and visualized using Geneious v6.1.7 created by Biomatters. Available from http://www.geneious.com/. Nucleotide alignments were performed using Geneious Alignment with default settings. For protein alignments, Clustal Omega (Version 1.2.1) was used with default settings, except order changed from aligned to input [18].

For phylogenetic analysis, the sequences were first aligned using the Clustal W program built into Geneious. Phylogenetic trees were constructed using the Geneious Tree Builder program, which uses the neighbour-joining method [31]. A 929 bp nucleotide fragment was used for the phylogenetic analysis of 16S rDNA sequences, while a 315 amino acid sequence alignment was used for phylogenetic analysis of the prenyltransferase. The outgroup was constituted by the distantly related cyanobacterium *Synechocystis sp*. for 16S rDNA analysis.

### PCR and sequencing reactions

A 50 μL PCR reaction mixture contained 10 pmol of specific forward and reverse primer (Additional file 11) (Geneworks, Australia), 1× PCR Buffer (KAPA Biosystems), 2.5 mM MgCl_2_, 1 pmol dNTPs (Fisher Biotec), 1 U of KapaTaq polymerase (KAPA Biosystems) and 50 ng of gDNA template. *Pfu* DNA polymerase (Sigma) was used in addition to KapaTaq at a ratio of 1:10 (v/v). Hotstart PCR was performed by first heating the samples to 95°C. Thermal cycling was then performed with a 5 min denaturation cycle at 95°C, followed by 30 cycles of 95°C for 30 sec, 55°C for 30 sec and 72°C for 1 min per 1 kb. Thermal cycling was concluded with a final extension at 72°C for 7 min. PCR products were visualized in 1% agarose gels in TAE buffer and single bands were gel extracted and purified using the QIAquick spin gel extraction kit (QIAGEN). Single sequencing reactions were submitted to the Ramaciotti Centre for Genomics at the University of New South Wales.

### Gene cloning for heterologous expression

The pJexpress411-T7-kan plasmids (with *C-* terminal His_6_-tag) harboring the codon-optimized genes of *welI1, welI3, welP1* and *welH* from WI HT-29-1 were purchased (DNA2.0, Inc, USA). A recombinant plasmid harboring the *ssuE* gene was generated by amplification from *E. coli* K12 with primers that incorporated the restriction sites *Nde*I and *Hind*III [32]. Amplification products were cloned into the pCR2.1 vector for sequencing, before excision and cloning into the pET28b expression vector. The cloning step permitted the fusion of the *N*-terminus of *ssuE* to the His_6_-tag present within pET28b.

### Heterologous protein expression and purification

#### WelI1 and WelI3

A 50% (v/v) glycerol stock of BL21(DE3) transformed with the gene of interest was used to inoculate a flask containing 25 mL LB broth supplemented with 50 μg/mL kanamycin. The flask was incubated at 37°C with shaking at 180 rpm for 6-8 h. This culture was added to a flask containing 1 L of LB broth supplemented with 50 μg/mL kanamycin and incubated at 37°C until an OD_600_ of approximately 0.6 was obtained. The cells were then induced with 1 mM IPTG and grown at 16°C overnight. The cells were centrifuged at 6,084 × *g* for 10 min and frozen at -20°C. The cell pellet was thawed on ice and resuspended in 50 mM Tris buffer (pH 7.5) containing a cocktail of protease inhibitors (Sigma Aldrich), 0.2 mM TCEP, 250 mM NaCl, and 10% (v/v) glycerol. Lysozyme was added to a final concentration of 1 mg/ml and stirred until a viscous suspension was obtained. The sample was sonicated under the following cycle: [(10 s pulse + 1 s pause) × 5, 1 min cooling period] repeated five times and the cellular debris was removed by centrifugation at 57,000 × *g* for 1 h at 4°C.

#### WelP1

pJexpress411*wel*P1 was freshly transformed into BL21(DE3) cells. An individual colony was picked and protein expression was performed as outlined in Hillwig *et al.*[7] for protein expression. Recombinant WelP1 was purified via immobilized metal affinity chromatography using a pre-packed His GraviTrap column (GE Healthcare). Imidazole was removed *via* dialysis using SnakeSkin dialysis tubing (3.5 kDa cutoff) (Thermo Scientific, Rockford, USA) and concentrated using Ambicon Ultra filters. Purified protein was then snap-frozen and stored at -80°C.

#### WelH and SsuE

pJexpress411*wel*H was freshly transformed into BL21(DE3) cells, and a single colony was used to inoculate 50 mL of LB media supplemented with 50 μg/mL kanamycin. The flask was incubated at 37°C with shaking at 180 rpm for 7.5 h. 25 mL of this culture was then used to inoculate 1 L of LB media supplemented with 50 μg/mL kanamycin, and incubated at 37°C until an OD_600_ of approximately 0.6 was obtained. Protein expression was then induced with 1 mM IPTG and grown at 16°C overnight. The cells were then collected by centrifugation at 8,000 × *g* for 10 min at 4°C. Cell pellets were thawed on ice and resuspended in 50 mM Tris (pH 7.5), 0.5 mM PMSF, 250 mM NaCl and 10% (v/v) glycerol. Lysozyme was then added to a final concentration of 1 mg/mL. Once a viscous suspension was achieved, cells were lysed via sonication (5× 10 sec pulse with 1 sec pause, 1 min cooling period, repeated four times). The cellular debris was removed by centrifugation at 8,000 × *g* at 4°C for 30 min. The imidazole concentration of the soluble protein fraction was first adjusted to 10 mM. Purification was then performed using His GraviTrap column (GE Healthcare). After the soluble protein was run through the column, 50 mM Tris (pH 7.5), 10 mM imidazole, 250 mM NaCl and 10% glycerol was used to wash the column. The beads were then washed with increasing concentrations of imidazole to remove contaminating proteins (25 and 50 mM imidazole). WelH was then eluted from the column by addition of 10 mL of 50 mM Tris (pH 7.5), 100 mM imidazole, 250 mM NaCl and 10% glycerol and 10 mL of 50 mM Tris (pH 7.5), 250 mM imidazole, 250 mM NaCl and 10% glycerol. These fractions were then combined and dialyzed against 20 mM Tris (pH 7.5), 0.2 mM TCEP, 250 mM NaCl and 20% glycerol using SnakeSkin dialysis tubing (3.5 kDa cutoff) (Thermo Scientific, Rockford, USA). The protein was then snap frozen and stored at -80°C.

pET28b*ssuE* was also freshly transformed into BL21(DE3) cells and protein expression and purification was performed as outlined in Dorrestein *et al.*[32].

#### Enzymatic assay with cell lysates (WelI1 and WelI3)

Each cell lysate containing a protein of interest (WelI1 or WelI3) totaled approximately 10 mL (resulting from 1 L of culture). Assay components were mixed to a final reaction volume of 5 mL (1 mL WelI1 cell lysate, 1 mL WelI3 cell lysate, 25 mM Tris (pH 7.0), 150 mM NaCl, 0.8 mg/ml L-tryptophan, 0.8 mg/ml ribose-5-phosphate, 0.8 mg/ml α-ketoglutaric acid, 25 μM (ammonium iron(II) sulphate). Samples were then incubated for 16 h at 25°C and extracted with 1:1 isopropanol/hexanes. Following extraction, samples were analyzed by HPLC. A negative control was performed with *E. coli* BL21 (DE3) cell lysate hosting no plasmid.

#### WelP1, WelH and SsuE enzymatic assay

For WelP1 assay only, 1 mM mixture of *cis* and *trans* isomers of indole-isonitrile standard, 1 mM GPP, 0 and 5 mM MgCl_2,_ 100 mM Tris (pH 7.5), 2 mM DTT and 15 μg of WelP1. The assay was incubated at 26 and 30°C for 1 and 16 h.

1 μM WelP1, 1 μM WelH and 3 μM SsuE was added to a 500 μL reaction containing 1 mM mixture of *cis* and *trans* isomers of indole-isonitrile standard, 1 mM GPP, 5 mM MgCl_2_, 20 mM Tris (pH 7.5), 25 mM NaCl, 2.4 mM β-nicotinamide adenine dinucleotide, reduced dipotassium salt (NADH) and 20 μM flavin adenine dinucleotide disodium salt hydrate (FAD). The enzymatic assay was incubated at 26°C for both 3 h and 5 h and 30°C for 3 h.

Attempts to optimize this assay included altering the concentration of enzymes (1-2 μM WelP1 and WelH, 3-6 μM SsuE), the concentration of the starting compounds (0.5 mM mixture of *cis* and *trans* isomers of indole-isonitrile and 0.5 mM GPP), the concentration of NaCl (0 and 25 mM), the concentration of NADH (2.4 and 10 mM) and the addition of 5% glycerol at 26 and 30°C for 15 h.

WelH and SsuE were also tested against L-tryptophan and GPP with and without WelP. In this assay, 1 μM WelH and 3 μM SsuE was added to a 500 μL reaction containing either 1 mM L-tryptophan or 1 mM GPP, 20 mM Tris (pH 7.5), 25 mM NaCl, 2.4 mM NADH and 20 μM FAD. 0 and 1 μM WelP was also added. The enzymatic assay was incubated at both 26 and 30°C for 3 h and extracted as per WelP1, WelH and SsuE assay above.

We also attempted the assay using the isonitrile proteins WelI1 and WelI3 with WelP1. 60 ng WelI1, 60 ng WelI3, 3 nM WelP1 was added to 0.8 mg/mL L-tryptophan, 1 mM GPP, 0.8 mg/mL D-ribose-5-phosphate disodium salt hydrate, 0.8 mg/mL α-ketoglutarate, 25 μM iron ammonium sulphate hexahydrate, 25 mM Tris (pH 7.5), 150 mM NaCl, 5 mM MgCl_2_, in 500 uL reaction. The reaction was performed for 16 h at 26°C. The assay was also attempted using 3 nM WelH and 9 nM SsuE.

All enzymatic products were extracted with three volumes of 1% acetic acid in ethyl acetate twice, dried, redissolved in 600 μL of methanol, and filtered through 0.2 μm PVDF filters (Grace Davison Discovery Sciences, USA). The extracted products were analyzed at the UWS MS Facility, Australia. Mass spectrometric analysis was undertaken using a Waters Xevo TQ-MS triple quadrupole instrument. Methanolic solutions were directly infused at 5 μL/min and data for each sample was recorded over the range *m/z* 10-500 in MS1 mode for a period of 10 min. Positive ion spectra were recorded with the following parameters: capillary voltage 3.50 kV; cone voltage 25 V; desolvation temperature 150°C; desolvation gas flow 400 L/hr; cone gas flow 0 L/hr. Negative ion spectra were recorded with the following parameters: capillary voltage 3.00 kV; cone voltage 20 V; desolvation temperature 300°C; desolvation gas flow 550 L/hr; cone gas flow 5 L/hr.

#### Indole-isonitrile metabolite extraction from FS ATCC43239 and FA UTEX1903

Fresh biomass was collected from FS ATCC43239 and FA UTEX1903 cultures by centrifugation at 3,500 × *g* for 10 min and then extracted with 60% (v/v) aqueous acetonitrile for 24 h at 4°C. Acetonitrile was removed using rotary evaporation and the collected aqueous layer was extracted with three equal volumes of ethyl acetate. After removal of ethyl acetate *in vacuo*, residue was stored at -80°C, until subjected to fractionation. For purification, silica gel was quenched with 0.5% triethyl amine in ethyl acetate:hexane mixture (5:94.5). Fractions were collected in 5%, 10%, 20%, 30%, 40% and 50% ethyl acetate/hexane gradient. Fractions with indole-isonitrile co-eluted at 40% ethyl acetate/hexane (alongside few other metabolites). Collected fractions were further purified by silica gel (quenched with 5% triethyl amine) chromatography and the fractions containing indole-isonitrile were analyzed through LCMS and HRMS.

#### LC-MS, HRESI-MS and HPLC Analyses

Accurate LC-MS data of cyanobacterial extracts were recorded with a Waters Acquity I-Class UPLC system and a Waters Synapt G2 HDMS mass spectrometer. High-resolution electrospray ionization-mass spectrometry (HRESI-MS) data for synthetic compounds and cyanobacterial extracts were obtained by direct infusion of methanolic solutions on a Waters Synapt HDMS QTOF mass spectrometer (Waters Corporation, Milford, MA). HPLC analyses for synthetic intermediates were performed using a Shimadzu LC-20-AT Series separations module equipped with Shimadzu SPD-M20A PDA (photo diode array) multiple wavelength detectors (180 nm-800 nm). For indole-isonitrile compounds, UV detector was set at 310 nm with a 5 nm slit-width. The overall system, CBM-20 was controlled using LC Solutions software. Raw data was plotted using Origin® software program after exporting absorbance data as an ASCII-formatted file. Analytical separations of stereoisomers (of *cis* and *trans*) mixtures were carried out on Daicel® (normal phase) AS chiral column. A 10% isopropanol/ 90% hexanes mixture was used as elution medium with a flow rate of 1 mL/min in an isocratic mode. Individual retention times for indole-isonitriles are reported along with analytical data for each isomer.

#### Synthesis and spectroscopic analysis of indole-isonitrile

Anhydrous tetrahydrofuran was obtained from mBraun solvent purification system (A2 alumina). Reactions were monitored by thin-layer chromatography (TLC) on silica gel plates (60 F_254_) with a fluorescent indicator, and independently visualized with UV light. Preparatory thin-layer chromatography (TLC) was performed on glass plates (7.5 × 2.5 and 7.5 × 5.0 cm) pre-coated glass plates coated with 60 Å silica gel (Whatman). Separations of isonitrile intermediates were carried out using flash chromatography (Silica gel grade: 200-400 mesh, 40-63 μm) at medium pressure (20 psi). NMR spectra were recorded at 400 MHz in CDCl_3_ and chemical shift values (δ) are reported in ppm. ^1^H NMR spectra are reported in parts per million (δ) relative to the residual (indicated) solvent peak. Data for ^1^H NMR are reported as follows: chemical shift (δ ppm), multiplicity (s = singlet, brs = broad singlet, d = doublet, t = triplet, q = quartet, ddd = double double doublet, m = multiplet, cm = complex multiplet), integration, and coupling constants in Hz. ^13^C NMR spectra were obtained on 400 MHz spectrometers (100 MHz actual frequency) and are reported in parts per million (δ) relative to the residual (indicated) solvent peak. High-resolution mass spectrometry (HRMS) data were obtained on spectrometer with a quadrupole analyzer.

### Synthesis of *cis* and *trans* isomers of indole-isonitrile

#### 3-Indolecarbaldehyde synthesis

To 7 mL of *N, N*-dimethylformamide (DMF), 1 mL of phosphorus oxychloride (POCl_3_) was added drop-wise at 0°C. The mixture was stirred for 20 min, after which 3 mL of 10 mmol indole in DMF was added dropwise to the mixture. After the mixture was stirred at 35°C for 1 h, crushed ice was added, followed by 20% aq. sodium hydroxide (NaOH) and the mixture was refluxed for 6 h maintained at 35°C. On cooling, the mixture was poured into ice water, and the precipitated product was collected, washed by water, and dried. 3-indolecarbaldehyde was the sole product and was isolated in 84% yield. This product was sufficiently pure for subjection to isonitrile formation step as shown from its ^1^H NMR spectrum. ^1^H NMR (400 MHz, DMSO-*d*6): δ 9.97 (s, 1H), 8.33 (s, 1H), 8.13 (d, *J* = 7.6 Hz, 1H), 7.55 (d, *J* = 7.2 Hz, 1H), 7.31–7.24 (m, 2H).

#### Synthesis of indole-isonitrile (3-(2-isocyanovinyl)indole)

A 5 mL THF solution containing 584 mg (3.3 mmol, 1.1 equiv.) of diethyl (isocyanomethyl) phosphonate was added drop wise to a stirred solution containing 839 mg (4.57 mmol, 1.5 equiv.) of sodium bis (trimethylsilyl)amide in 5 mL of THF at - 78°C. The resulting mixture was stirred for 15 min and then treated with a solution of 436 mg (3.0 mmol, 1.0 equiv.) of 3-indolecarbaldehyde in 30 mL of THF. The solution was allowed to warm to 4°C and allowed to stir for an additional 48 h. 198 mg (3.3 mmol) of acetic acid in 1.5 mL of THF was added to quench the reaction. The solvent was removed in vacuo, the residue was dissolved in 30 mL of ethyl acetate, washed with 15 mL of 0.1 M phosphate buffer (pH = 7.2), then with 15 mL of H_2_O and the resulting organic layer was dried on a bed of MgSO_4_. Collected organic layer was evaporated to obtain the crude product which upon purification through chromatography (silica gel) eluting with a gradient of 10-12% ethyl acetate in hexane yielded a mixture of *trans* (196 mg, 45%) and *cis* (106 mg, 25%) indole-isonitrile in a 3:2 ratio as indicated by ^1^H NMR analysis and in 70% overall yield.

#### *trans* indole-isonitrile synthesis

^1^H NMR (400 MHz, CDCl_3_) δ 8.35 (brs, 1H), 7.69 (d, 7.9 Hz, 1H), 7.44-7.40 (m, 1H), 7.35 (d, *J* = 2.6 Hz, 1H), 7.32-7.21 (m, 2H), 7.14 (d, *J* = 14.2 Hz, 1H), 6.36 (d, *J* = 14.2 Hz, 1H). ^13^C NMR (100 MHz, CDCl_3_) δ 163.3, 137.0, 130.3, 126.4, 124.8, 123.6, 121.6, 120.1, 112.0, 111.3, 107.3 (Additional file 5).

#### *cis* indole-isonitrile synthesis

^1^H NMR (400 MHz, CDCl_3_) δ 8.56 (brs, 1H), 8.15 (d, 2.8 Hz, 1H), 7.68 (d, 7.9 Hz, 1H), 7.44 (d, *J* = 7.9 Hz, 1H), 7.32-7.20 (m, 2H), 6.84-6.75 (m, 1H), 5.75 (d, *J* = 8.8 Hz, 1H). ^13^C NMR (100 MHz, CDCl_3_) δ 169.1, 135.2, 126.9, 126.5, 124.2, 123.4, 121.1, 118.2, 111.6, 110.3, 104.6 (Additional file 5).

The R_*f*_ value (40% EtOAC in hexanes) for the *cis* isomer of isonitrile is: 0.52, and the R_*f*_ value (40% EtOAC in hexanes) for the *trans* isomer of isonitrile is: 0.36. These R_*f*_ values were applied for identification and comparison of isonitriles in metabolic extracts from FS ATCC43239 and FA UTEX1903 cyanobacterial strains as described in this study. The ^1^H NMR spectra and ^13^C NMR data of the synthesized standard matched those reported by Hoppe and Schollkopf [33].

#### Nucleotide sequence accession numbers

The nucleotide sequence of the gene clusters were deposited to NCBI GenBank under the following accession numbers: KJ742064 for FS ATCC43239, JK742065 for FA UTEX1903, KJ767018 for WI HT-29-1 and KJ767017 for HW IC-52-3. The nucleotide sequence of the 16S ribosomal RNA gene was also deposited to NCBI GenBank under the following accession numbers: KJ768872 for FS ATCC43239, KJ768871 for FA UTEX1903, KJ767016 for WI HT-29-1 and KJ767019 for HW IC-52-3.

## Competing interests

The authors declare that they have no competing interests.

## Authors’ contributions

MCM and RV designed the overall project. MLM and MCM sequenced the genomes of WI HT-29-1 and HW IC-52-3. DS and RV sequenced the genomes of FA UTEX1903 and FS ATCC43239. MLM and DS jointly contributed to identification and functional assignment of the gene clusters. MLM and LG jointly contributed to protein expression of WelP1, WelH and SsuE. BMB contributed to the functional assignment, protein expression and reconstitution of WelI1 and WelI3. DS contributed to chemical synthesis and characterization of cyanobacterial extracts. MCM, LG and RV edited the final version of the manuscript drafted jointly by MLM, DS and BMB. All authors read and approved the final manuscript.

## Additional files

## Supplementary Material

Additional file 1: **BLASTx analysis of gene clusters analyzed in this study.Table S1.** The *wel* gene cluster in *Westiella intricata* UH strain HT-29-1. **Table S2.** The *wel* gene cluster in *Hapalosiphon welwitschii* UH strain IC-52-3. **Table S3.** The *hpi* gene cluster in *Fischerella sp.* ATCC 43239. **Table S4.** The *amb* gene cluster in *Fischerella ambigua* UTEX 1903 from this study. **Table S5.** The *hpi* gene cluster in *Fischerella sp.* PCC 9339. **Table S6.** The *wel* gene cluster in *Fischerella sp.* PCC 9431. **Table S7.** The *wel* gene cluster in *Fischerella muscicola* SAG 1427-1. Click here for file

Additional file 2: Phylogenetic analysis of HpiP1/AmbP1/WelP1 enzyme. Click here for file

Additional file 3: Sequence alignment and identification of conserved motifs from isonitrile proteins I1and I2. Click here for file

Additional file 4: Sequence alignment of isonitrile protein I3 with IsnB and PvcB. Click here for file

Additional file 5: ^**1**^**H and**^**13**^**C NMR and HRMS spectra for chemically synthesized** ***cis*** **and** ***trans*** **indole-isonitriles.** Click here for file

Additional file 6: LC-ESI-MS spectrum for enzyme-catalyzed indole-isonitrile biosynthesis product. Click here for file

Additional file 7: HRESI-MS and MS peaks from LC-MS spectra for chemically synthesized indole-isonitrile and cyanobacterial extracts from FS ATCC43239 and FA UTEX1903. Click here for file

Additional file 8: Sequence identity of all oxygenase proteins. Click here for file

Additional file 9: Sequence alignment and identification of motifs from Reiske-type oxygenases. Click here for file

Additional file 10: Sequence identity of all unknown proteins with domain of unknown function. Click here for file

Additional file 11: Specific primers used in this study. Click here for file
