# Supplementary material for: Comparative analysis of hapalindole, ambiguine and welwitindolinone gene clusters and reconstitution of indole-isonitrile biosynthesis from cyanobacteria
Source: BMC Microbiol. 2014 Aug 1;14:213. doi: 10.1186/s12866-014-0213-7 (PMC4236562; doi:10.1186/s12866-014-0213-7)
Supplement: Additional file 1: — BLASTx analysis of gene clusters analyzed in this study.Table S1. The wel gene cluster in Westiella intricata UH strain HT-29-1. Table S2. The wel gene cluster in Hapalosiphon welwitschii UH strain IC-52-3. Table S3. The hpi gene cluster in Fischerella sp. ATCC 43239. Table S4. The amb gene cluster in Fischerella ambigua UTEX 1903 from this study. Table S5. The hpi gene cluster in Fischerella sp. PCC 9339. Table S6. The wel gene cluster in Fischerella sp. PCC 9431. Table S7. The wel gene cluster in Fischerella muscicola SAG 1427-1. [file s12866-014-0213-7-S1.docx]

# Additional File 1, Table S1: **BLASTx analysis of the w**elwitindolinone (*wel*) gene cluster in *Westiella intricata* UH strain HT-29-1:

| **Gene** | **Size^*^ (aa)** | **Top BLASTx**  **(nucleotide-protein) hit** | **Organism** | **Accession number** | **Identity/**  **Similarity** |
| --- | --- | --- | --- | --- | --- |
| *welO15* | 290 | AmbO5 | *Fischerella ambigua* UTEX 1903 | AHB62755.1 | 78/88 |
| *welM1* | 347 | Hypothetical protein | *Fischerella* sp*.* PCC 9339 | WP_017307678.1 | 55/72 |
| *welU8* | 226 | AmbU3 | *Fischerella ambigua* UTEX 1903 | AHB62758.1 | 61/72 |
| *welO14* | 359 | AmbO1 | *Fischerella ambigua* UTEX 1903 | AHB62763.1 | 68/81 |
| *welO13* | 362 | AmbO3 | *Fischerella ambigua* UTEX 1903 | AHB62761.1 | 69/81 |
| *welO12* | 357 | HpiO8 | *Fischerella* sp*.* PCC 9339 | WP_017308631.1 | 72/83 |
| *welO11* | 359 | HpiO8 | *Fischerella* sp*.* PCC 9339 | WP_017308631.1 | 75/85 |
| *welU6* | 228 | HpiU6 | *Fischerella* sp*.* PCC 9339 | WP_017308630.1 | 93/96 |
| *welD4* | 400 | HpiD4 | *Fischerella* sp*.* PCC 9339 | WP_01738629.1 | 96/98 |
| *welP2* | 334 | HpiP2 | *Fischerella* sp*.* PCC 9339 | WP_01738628.1 | 89/93 |
| *welR1* | 234 | HpiR1/ AmbR1 | *Fischerella* sp*.* PCC 9339 / *Fischerella ambigua* UTEX 1903 | WP_01738626.1 / AHB62767.1 | 92/96 |
| *welR2* | 243 | HpiR2 | *Fischerella* sp*.* PCC 9339 | WP_01738625.1 | 94/95 |
| *welR3* | 363 | WelR3 | *Fischerella muscicola* | WP_016862237.1 | 87/93 |
| *welC3* | 214 | HpiC3 | *Fischerella* sp*.* PCC 9339 | WP_017308609.1 | 96/98 |
| *welT5* | 365 | AmbT5 | *Fischerella ambigua* UTEX 1903 | AHB62782.1 | 93/95 |
| *welT4* | 415 | WelT4 | *Fischerella muscicola* | WP_016862234.1 | 97/99 |
| *welT3* | 274 | WelT3 | *Fischerella muscicola* | WP_016862233.1 | 91/94 |
| *welT2* | 283 | HpiT2 | *Fischerella* sp*.* PCC 9339 | WP_017308613.1 | 90/96 |
| *welT1* | 734 | AmbT1 | *Fischerella ambigua* UTEX 1903 | AHB62778.1 | 91/95 |
| *welC2* | 301 | AmbC2 | *Fischerella ambigua* UTEX 1903 | AHB62777.1 | 98/98 |
| *welO18* | 482 | WelO15 | *Fischerella muscicola* | WP_016862229.1 | 99/99 |
| *welO19* | 358 | WelO16 | *Fischerella muscicola* | WP_016862228.1 | 98/98 |
| *welD3* | 408 | HpiD3 / AmbD3 | *Fischerella* sp*.* PCC 9339 / *Fischerella ambigua* UTEX 1903 | WP_017308616.1 / AHB62776.1 | 99/99 |
| *welD2* | 647 | AmbD2 | *Fischerella ambigua* UTEX 1903 | AHB62775.1 | 97/98 |
| *welP1* | 308 | WelP1 | *Fischerella muscicola* | WP_016862225.1 | 97/98 |
| *welI3* | 273 | HpiI3 | *Fischerella* sp*.* PCC 9339 | WP_017308619.1 | 96/97 |
| *welI2* | 330 | AmbI2 | *Fischerella ambigua* UTEX 1903 | AHB62772.1 | 96/98 |
| *welI1* | 319 | HpiI1 / AmbI1 | *Fischerella* sp*.* PCC 9339 / *Fischerella ambigua* UTEX 1903 | WP_017308621.1 / AHB62771.1 | 94/98 |
| *welD1* | 414 | HpiD1 / AmbD1 | *Fischerella* sp*.* PCC 9339 / *Fischerella ambigua* UTEX 1903 | WP_017308622.1 / AHB62770.1 | 95/98 |
| *welC1* | 192 | HpiC1 / AmbC1 | *Fischerella* sp*.* PCC 9339 / *Fischerella ambigua* UTEX 1903 | WP_017308623.1 / AHB62769.1 | 93/96 |
| *welM2* | 256 | WelM2 | *Fischerella muscicola* | WP_016862218.1 | 83/92 |
| *orf1* | 359 | Orf1 | *Fischerella muscicola* | WP_016862217.1 | 96/98 |
| *orf2* | 56 | Hypothetical protein | *Fischerella* sp*.* PCC 9339 | WP_017308491.1 | 93/94 |
| *orf3* | 903 | Hypothetical protein Npun_F2218 | *Nostoc punctiforme* PCC 73102 | YP_001865757.1 | 87/93 |
| *welH* | 542 | Hypothetical protein | *Fischerella* sp*.* PCC 9339 | WP_017308202.1 | 72/85 |
| *welM3* | 279 | Hypothetical protein | *Fischerella* sp*.* JSC-11 | WP_009458912.1 | 96/98 |
| *welE4* | 105 | Small multidrug resistance protein | *Fischerella* sp*.* JSC-11 | WP_009458415.1 | 77/88 |
| *orf4* | 130 | Hypothetical protein | *Oscillatoria* sp. PCC 10802 | WP_017715310.1 | 47/66 |
| *orf5* | 252 | Short chain dehydrogenase | *Gloeobacter violaceus* PCC 7421 | NP_925784.1 | 62/80 |
| *welO16* | 382 | Hypothetical protein | *Calothrix* sp. PCC 7130 | WP_019496741.1 | 57/74 |
| *orf6* | 237 | Hypothetical protein | *Calothrix* sp. PCC 7103 | WP_019496743.1 | 50/70 |
| *orf7* | 277 | Hypothetical protein | *Fischerella* sp*.* PCC 9339 | WP_017307918.1 | 81/89 |
| *welO17* | 518 | Hypothetical protein | *Scytonema hofmanni* | WP_017741725.1 | 89/94 |
| *orf8* | 171 | DoxX protein | *Nostoc* sp. PCC 7524 | YP_007075410.1 | 91/95 |
| *orf9* | 471 | NADH dehydrogenase, FAD-containing subunit | *Nostoc* sp. PCC 7524 | YP_007075409.1 | 88/94 |
| *orf10* | 309 | Tail collar domain-containing protein | *Herpetosiphon aurantiacus* DSM 785 | YP_001544744.1 | 59/75 |
| *welS1* | 326 | Transposase IS4 family protein | *Pseudanabaena biceps* PCC 7429 | ZP_21067860.1 | 60/75 |
| ^*^ Size of encoded protein | | | | | |

Additional File 1, Table S2: BLASTx analysis of the welwitindolinone (*wel*) gene cluster in *Hapalosiphon welwitschii* UH strain IC-52-3:

| **Gene** | **Size^*^ (aa)** | **Top BLASTx**  **(nucleotide-protein) hit** | **Organism** | **Accession number** | **Identity/**  **Similarity** |
| --- | --- | --- | --- | --- | --- |
| *welO15* | 290 | AmbO5 | *Fischerella ambigua* UTEX 1903 | AHB62755.1 | 80/90 |
| *welM1* | 347 | Hypothetical protein | *Fischerella* sp*.* PCC 9339 | WP_017307678.1 | 56/72 |
| *welU8* | 226 | AmbU3 | *Fischerella ambigua* UTEX 1903/ | AHB62758.1 | 61/71 |
| *welO14* | 359 | AmbO1 | *Fischerella ambigua* UTEX 1903/ | AHB62763.1 | 68/80 |
| *welO13* | 362 | AmbO3 | *Fischerella ambigua* UTEX 1903/ | AHB62761.1 | 69/80 |
| *welO12* | 357 | HpiO8 | *Fischerella* sp*.* PCC 9339 | WP_017308631.1 | 72/83 |
| *welO11* | 328 | HpiO8 | *Fischerella* sp*.* PCC 9339 | WP_017308631.1 | 74/85 |
| *welU6* | 227 | HpiU6 | *Fischerella* sp*.* PCC 9339 | WP_017308630.1 | 91/93 |
| *welU7* | 228 | AmbU4 | *Fischerella ambigua* UTEX 1903 | AHB62757.1 | 78/88 |
| *welD4* | 400 | HpiD4 | *Fischerella* sp*.* PCC 9339 | WP_01738629.1 | 95/98 |
| *welP2* | 334 | AmbP2 | *Fischerella ambigua* UTEX 1903 | AHB62766.1 | 89/93 |
| *welR1* | 234 | HpiR1/ AmbR1 | *Fischerella* sp*.* PCC 9339 / *Fischerella ambigua* UTEX 1903 | WP_01738626.1 / AHB62767.1 | 92/96 |
| *welR2* | 243 | AmbR2 | *Fischerella ambigua* UTEX 1903 | AHB62768.1 | 94/96 |
| *welR3* | 363 | WelR3 | *Fischerella muscicola* | WP_016862237.1 | 87/93 |
| *welC3* | 214 | HpiC3 | *Fischerella* sp*.* PCC 9339 | WP_017308609.1 | 96/98 |
| *welT5* | 365 | AmbT5 | *Fischerella ambigua* UTEX 1903 | AHB62782.1 | 93/95 |
| *welT4* | 415 | WelT4 | *Fischerella muscicola* | WP_016862234.1 | 96/98 |
| *welT3* | 274 | WelT3 | *Fischerella muscicola* | WP_016862233.1 | 91/94 |
| *welT2* | 283 | HpiT2 | *Fischerella* sp*.* PCC 9339 | WP_017308613.1 | 91/95 |
| *welT1* | 734 | AmbT1 | *Fischerella ambigua* UTEX 1903 | AHB62778.1 | 91/95 |
| *welC2* | 301 | AmbC2 | *Fischerella ambigua* UTEX 1903 | AHB62777.1 | 97/98 |
| *welD3* | 408 | HpiD3 / AmbD3 | *Fischerella* sp*.* PCC 9339 / *Fischerella ambigua* UTEX 1903 | WP_017308616.1 / AHB62776.1 | 99/99 |
| *welD2* | 647 | AmbD2 | *Fischerella ambigua* UTEX 1903 | AHB62775.1 | 97/98 |
| *welP1* | 308 | HpiP1 / AmbP1 | *Fischerella* sp*.* PCC 9339 / *Fischerella ambigua* UTEX 1903 | WP_017308618.1 / AHB62774.1 | 96/97 |
| *welI3* | 273 | HpiI3 | *Fischerella* sp*.* PCC 9339 | WP_017308619.1 | 97/98 |
| *welI2* | 331 | AmbI2 | *Fischerella ambigua* UTEX 1903 | AHB62772.1 | 96/97 |
| *welI1* | 319 | HpiI1 / AmbI1 | *Fischerella* sp*.* PCC 9339 / *Fischerella ambigua* UTEX 1903 | WP_017308621.1 / AHB62771.1 | 94/98 |
| *welD1* | 406 | HpiD1 / AmbD1 | *Fischerella* sp*.* PCC 9339 / *Fischerella ambigua* UTEX 1903 | WP_017308622.1 / AHB62770.1 | 96/98 |
| *welC1* | 192 | HpiC1 / AmbC1 | *Fischerella* sp*.* PCC 9339 / *Fischerella ambigua* UTEX 1903 | WP_017308623.1 / AHB62769.1 | 94/97 |
| *welM2* | 256 | WelM2 | *Fischerella muscicola* | WP_016862218.1 | 84/92 |
| *orf1* | 359 | Orf1 | *Fischerella muscicola* | WP_016862217.1 | 97/98 |
| *orf2* | 56 | Hypothetical protein | *Fischerella* sp*.* PCC 9339 | WP_017308491.1 | 93/94 |
| *orf3* | 903 | Hypothetical protein Npun_F2218 | *Nostoc punctiforme* PCC 73102 | YP_001865757.1 | 87/93 |
| *welH* | 542 | Hypothetical protein | *Fischerella* sp*.* PCC 9339 | WP_017308202.1 | 71/85 |
| *welM3* | 279 | Hypothetical protein | *Fischerella* sp*.* JSC-11 | WP_009458912.1 | 93/97 |
| *welE4* | 105 | Small multidrug resistance protein | *Fischerella* sp*.* JSC-11 | WP_009458415.1 | 79/90 |
| *orf4* | 136 | Hypothetical protein | *Oscillatoria* sp. PCC 10802 | WP_017715310.1 | 46/66 |
| *orf5* | 252 | Short chain dehydrogenase | *Gloeobacter violaceus* PCC 7421 | NP_925784.1 | 63/80 |
| *welO16* | 382 | Hypothetical protein | *Calothrix* sp*.* PCC 7130 | WP_019496741.1 | 57/74 |
| *orf6* | 237 | Hypothetical protein | *Calothrix* sp. PCC 7103 | WP_019496743.1 | 50/70 |
| *orf7* | 277 | Hypothetical protein | *Fischerella* sp*.* PCC 9339 | WP_017307918.1 | 80/89 |
| *welO17* | 518 | Hypothetical protein | *Scytonema hofmanni* | WP_017741725.1 | 89/94 |
| *orf8* | 171 | DoxX protein | *Nostoc* sp. PCC 7524 | YP_007075410.1 | 91/95 |
| *orf9* | 471 | NADH dehydrogenase, FAD-containing subunit | *Nostoc* sp. PCC 7524 | YP_007075409.1 | 88/94 |
| *orf10* | 309 | Tail collar domain-containing protein | *Herpetosiphon aurantiacus* DSM 785 | YP_001544744.1 | 59/75 |
| ^*^ Size of encoded protein | | | | | |

Additional File 1, Table S3: BLASTx analysis of the hapalindole (*hpi*) gene cluster in *Fischerella* sp*.* ATCC 43239:

| **Gene** | **Size^*^ (aa)** | **Top BLASTx**  **(nucleotide-protein) hit** | **Organism** | **Accession number** | **Identity/**  **Similarity** |
| --- | --- | --- | --- | --- | --- |
| *orf1* | 796 | Orf1 | *Fischerella* sp*.* PCC 9339 | WP_017308608.1 | 99/99 |
| *hpiC3* | 214 | AmbC3 | *Fischerella ambigua* UTEX 1903 | AHB62783.1 | 99/99 |
| *hpiT5* | 365 | AmbT5 | *Fischerella ambigua* UTEX 1903 | AHB62782.1 | 99/99 |
| *hpiT4* | 415 | AmbT4 | *Fischerella ambigua* UTEX 1903 | AHB62781.1 | 99/99 |
| *hpiT3* | 270 | AmbT3 | *Fischerella ambigua* UTEX 1903 | AHB62780.1 | 1000/100 |
| *hpiT2* | 287 | AmbT2 | *Fischerella ambigua* UTEX 1903 | AHB62779.1 | 99/99 |
| *hpiS1* | 315 | Hypothetical protein | *Microcoleus vaginatus* | WP_006631927.1 | 53/73 |
| *hpiT1* | 734 | AmbT1 | *Fischerella ambigua* UTEX 1903 | AHB62778.1 | 99/99 |
| *hpiC2* | 301 | AmbC2 | *Fischerella ambigua* UTEX 1903 | AHB62777.1 | 100/100 |
| *hpiD3* | 408 | HpiD3 / AmbD3 | *Fischerella* sp*.* PCC 93393 / *Fischerella ambigua* UTEX 1903 | AHB62776.1 / WP_017308616 | 99/99 |
| *hpiD2* | 647 | AmbD2 | *Fischerella ambigua* UTEX 1903 | AHB62775.1 | 99/99 |
| *hpiP1* | 309 | HpiP1 / AmbP1 | *Fischerella* sp*.* PCC 93393 / *Fischerella ambigua* UTEX 1903 | WP_017308618.1 / AHB62774.1 | 98/98 |
| *hpiI3* | 273 | HpiI3 | *Fischerella* sp*.* PCC 93393 | WP_017308619.1 | 98/98 |
| *hpiI2* | 330 | AmbI2 | *Fischerella ambigua* UTEX 1903 | AHB62772.1 | 98/99 |
| *hpiI1* | 319 | HpiI1 / AmbI1 | *Fischerella* sp*.* PCC 9339 / *Fischerella ambigua* UTEX 1903 | WP_017308621.1 / AHB62771.1 | 99/99 |
| *hpiD1* | 414 | HpiD1 / AmbD1 | *Fischerella* sp*.* PCC 9339 / *Fischerella ambigua* UTEX 1903 | WP_017308622.1 / AHB62770.1 | 99/99 |
| *hpiC1* | 192 | HpiC1 / AmbC1 | *Fischerella* sp*.* PCC 9339 / *Fischerella ambigua* UTEX 1903 | WP_017308623.1 / AHB62769.1 | 98/98 |
| *hpiR2* | 243 | AmbR2 | *Fischerella ambigua* UTEX 1903 | AHB62768.1 | 100/100 |
| *hpiR1* | 232 | HpiR1 / AmbR1 | *Fischerella* sp*.* PCC 9339 / *Fischerella ambigua* UTEX 1903 | WP_017308626.1 / AHB62767.1 | 100/100 |
| *hpiP2* | 346 | AmbP2 | *Fischerella ambigua* UTEX 1903 | AHB62766.1 | 99/99 |
| *hpiD4* | 400 | HpiD4 | *Fischerella* sp*.* PCC 9339 | WP_017308629.1 | 99/99 |
| *hpiU6* | 228 | HpiU6 | *Fischerella* sp*.* PCC 9339 | WP_017308630.1 | 99/99 |
| *hpiO8* | 358 | HpiO8 | *Fischerella* sp*.* PCC 9339 | WP_017308631.1 | 100/100 |
| *orf2* | 336 | Hypothetical protein | *Fischerella* sp*.* PCC 9339 | WP_017308632.1 | 99/99 |
| *hpiU1* | 243 | HpiU1 | *Fischerella* sp*.* PCC 9339 | WP_017308633.1 | 98/99 |
| *hpiU2* | 223 | AmbU2 | *Fischerella ambigua* UTEX 1903 | AHB62759.1 | 100/100 |
| *hpiU3* | 230 | AmbU3 | *Fischerella ambigua* UTEX 1903 | AHB62758.1 | 99/99 |
| *hpiO9* | 362 | AmbO3 | *Fischerella ambigua* UTEX 1903 | AHB62761.1 | 68/79 |
| *hpiU5* | 227 | AmbU4 | *Fischerella ambigua* UTEX 1903 | AHB62757.1 | 85/92 |
| *hpiO10* | 418 | FAD dependent oxidoreductase domain protein | *Leptospira kirschneri* | WP_016761647.1 | 51/64 |
| ^*^ Size of encoded protein | | | | | |

Additional File 1, Table S4: BLASTx analysis of the ambiguine (*amb*) gene cluster in *Fischerella ambigua* UTEX 1903 from this study:

| **Gene** | **Size^*^ (aa)** | **Top BLASTx**  **(nucleotide-protein) hit** | **Organism** | **Accession number** | **Identity/**  **Similarity** |
| --- | --- | --- | --- | --- | --- |
| *ambS1* | 197 | Hypothetical protein (transposase) | *Synechococcus* sp. PCC 7336 | WP_017327147.1 | 74/89 |
| *ambS2* | 102 | Hypothetical protein | *Fischerella* sp*.* PCC 9339 | WP_017307744.1 | 100/100 |
| *orf1* | 796 | Phosphoketolase | *Fischerella* sp*.* PCC 9339 | WP_01738608.1 | 99/99 |
| *ambC3* | 214 | AmbC3 | *Fischerella ambigua* UTEX 1903 | AHB62783.1 | 100/100 |
| *ambT5* | 365 | AmbT5 | *Fischerella ambigua* UTEX 1903 | AHB62782.1 | 100/100 |
| *ambT4* | 416 | AmbT4 | *Fischerella ambigua* UTEX 1903 | AHB62781.1 | 100/100 |
| *ambT3* | 270 | AmbT3 | *Fischerella ambigua* UTEX 1903 | AHB62780.1 | 100/100 |
| *ambT2* | 283 | AmbT2 | *Fischerella ambigua* UTEX 1903 | AHB62779.1 | 100/100 |
| *ambT1* | 734 | AmbT1 | *Fischerella ambigua* UTEX 1903 | AHB62778.1 | 100/100 |
| *ambC2* | 301 | AmbC2 | *Fischerella ambigua* UTEX 1903 | AHB62777.1 | 100/100 |
| *ambD3* | 408 | AmbD3 | *Fischerella ambigua* UTEX 1903 | AHB62776.1 | 100/100 |
| *ambD2* | 647 | AmbD2 | *Fischerella ambigua* UTEX 1903 | AHB62775.1 | 100/100 |
| *ambP1* | 309 | AmbP1 | *Fischerella ambigua* UTEX 1903 | AHB62774.1 | 100/100 |
| *ambI3* | 273 | AmbI3 | *Fischerella ambigua* UTEX 1903 | AHB62773.1 | 100/100 |
| *ambI2* | 330 | AmbI2 | *Fischerella ambigua* UTEX 1903 | AHB62772.1 | 100/100 |
| *ambI1* | 319 | AmbI1 | *Fischerella ambigua* UTEX 1903 | AHB62771.1 | 100/100 |
| *ambD1* | 406 | AmbD1 | *Fischerella ambigua* UTEX 1903 | AHB62770.1 | 100/100 |
| *ambC1* | 192 | AmbC1 | *Fischerella ambigua* UTEX 1903 | AHB62769.1 | 100/100 |
| *ambR2* | 244 | AmbR2 | *Fischerella ambigua* UTEX 1903 | AHB62768.1 | 100/100 |
| *ambR1* | 232 | AmbR1 | *Fischerella ambigua* UTEX 1903 | AHB62767.1 | 100/100 |
| *ambP2* | 346 | AmbP2 | *Fischerella ambigua* UTEX 1903 | AHB62766.1 | 100/100 |
| *ambD4* | 400 | AmbD4 | *Fischerella ambigua* UTEX 1903 | AHB62765.1 | 100/100 |
| *ambP3* | 322 | AmbP3 | *Fischerella ambigua* UTEX 1903 | AHB62764.1 | 100/100 |
| *ambO1* | 359 | AmbO1 | *Fischerella ambigua* UTEX 1903 | AHB62763.1 | 100/100 |
| *ambO2* | 360 | AmbO2 | *Fischerella ambigua* UTEX 1903 | AHB62762.1 | 100/100 |
| *ambO3* | 363 | AmbO3 | *Fischerella ambigua* UTEX 1903 | AHB62761.1 | 100/100 |
| *ambU1* | 239 | AmbU1 | *Fischerella ambigua* UTEX 1903 | AHB62760.1 | 100/100 |
| *ambU2* | 238 | AmbU2 | *Fischerella ambigua* UTEX 1903 | AHB62759.1 | 100/100 |
| *ambU3* | 230 | AmbU4 | *Fischerella ambigua* UTEX 1903 | AHB62757.1 | 100/100 |
| *ambU4* | 235 | AmbU3 | *Fischerella ambigua* UTEX 1903 | AHB62758.1 | 100/100 |
| *ambO4* | 285 | AmbO4 | *Fischerella ambigua* UTEX 1903 | AHB62756.1 | 100/100 |
| *ambO5* | 316 | AmbO5 | *Fischerella ambigua* UTEX 1903 | AHB62755.1 | 99/99 |
| *ambE1* | 388 | AmbE1 | *Fischerella ambigua* UTEX 1903 | AHB62754.1 | 100/100 |
| *ambE2* | 397 | AmbE2 | *Fischerella ambigua* UTEX 1903 | AHB62753.1 | 100/100 |
| *ambE3* | 151 | AmbE3 | *Fischerella ambigua* UTEX 1903 | AHB62752.1 | 100/100 |
| *ambO6* | 386 | HpiO6 | *Fischerella* sp*.* PCC 9339 | WP_017308644.1 | 100/100 |
| *orf-4* | 638 | Hypothetical protein | *Fischerella* sp*.* PCC 9339 | WP_017311018.1 | 99/99 |
| *orf-5* | 477 | Hypothetical protein | *Fischerella* sp*.* PCC 9339 | WP_017311020.1 | 99/99 |
| *ambO7* | 401 | Hypothetical protein | *Fischerella* sp*.* PCC 9339 | WP_017311021.1 | 100/100 |
| ^*^ Size of encoded protein | | | | | |

Additional File 1, Table S5: BLASTx analysis of the hapalindole (*hpi*) gene cluster in *Fischerella* sp*.* PCC 9339:

| **Gene** | **Size^*^ (aa)** | **IMG Gene ID** | **Top BLASTx**  **(nucleotide-protein) hit** | **Organism** | **Accession number** | **Identity/**  **Similarity** |
| --- | --- | --- | --- | --- | --- | --- |
| *hpiS1* | 181 | 2517064588 | DDE endonucleoase | *Coleofasciculus chthonoplastes* PCC 7420 | WP_006100142.1 | 58/75 |
| *hpiS2* | 102 | 2517064589 | Transposase | *Cyanothece* sp*.* CCY0110 | WP_008275513.1 | 47/70 |
| *orf1* | 796 | 2517064590 | Phosphoketolase | *Gloeocapsa* sp. PCC 7428 | YP_007127497.1 | 79/88 |
| *hpiC3* | 214 | 2517064591 | AmbC3 | *Fischerella ambigua* UTEX 1903 | AHB62783.1 | 97/99 |
| *hpiT5* | 365 | 2517064592 | AmbT5 | *Fischerella ambigua* UTEX 1903 | AHB62782.1 | 99/99 |
| *hpiT4* | 415 | 2517064593 | AmbT4 | *Fischerella ambigua* UTEX 1903 | AHB62781.1 | 98/99 |
| *hpiT3* | 270 | 2517064594 | AmbT3 | *Fischerella ambigua* UTEX 1903 | AHB62780.1 | 97/98 |
| *hpiT2* | 287 | 2517064595 | AmbT2 | *Fischerella ambigua* UTEX 1903 | AHB62779.1 | 94/97 |
| *hpiT1* | 734 | 2517064596 | AmbT1 | *Fischerella ambigua* UTEX 1903 | AHB62778.1 | 94/97 |
| *hpiC2* | 301 | 2517064597 | AmbC2 | *Fischerella ambigua* UTEX 1903 | AHB62777.1 | 96/97 |
| *hpiD3* | 408 | 2517064598 | AmbD3 | *Fischerella ambigua* UTEX 1903 | AHB62776.1 | 100/100 |
| *hpiD2* | 647 | 2517064599 | AmbD2 | *Fischerella ambigua* UTEX 1903 | AHB62775.1 | 99/99 |
| *hpiP1* | 309 | 2517064600 | AmbP1 | *Fischerella ambigua* UTEX 1903 | AHB62774.1 | 100/100 |
| *hpiI3* | 273 | 2517064602 | AmbI3 | *Fischerella ambigua* UTEX 1903 | AHB62773.1 | 99/100 |
| *hpiI2* | 331 | 2517064603 | AmbI2 | *Fischerella ambigua* UTEX 1903 | AHB62772.1 | 99/99 |
| *hpiI1* | 319 | 2517064604 | AmbI1 | *Fischerella ambigua* UTEX 1903 | AHB62771.1 | 100/100 |
| *hpiD1* | 406 | 2517064605 | AmbD1 | *Fischerella ambigua* UTEX 1903 | AHB62770.1 | 99/100 |
| *hpiC1* | 192 | 2517064606 | AmbC1 | *Fischerella ambigua* UTEX 1903 | AHB62769.1 | 100/100 |
| *hpiR2* | 243 | 2517064608 | AmbR2 | *Fischerella ambigua* UTEX 1903 | AHB62768.1 | 100/100 |
| *hpiR1* | 232 | 2517064609 | AmbR1 | *Fischerella ambigua* UTEX 1903 | AHB62767.1 | 100/100 |
| *hpiP2* | 346 | 2517064611 | AmbP2 | *Fischerella ambigua* UTEX 1903 | AHB62766.1 | 100/100 |
| *hpiD4* | 412 | 2517064612 | AmbD4 | *Fischerella ambigua* UTEX 1903 | AHB62765.1 | 96/98 |
| *hpiU6* | 228 | 2517064613 | AmbU3 | *Fischerella ambigua* UTEX 1903 | AHB62758.1 | 70/80 |
| *hpiO8* | 358 | 2517064614 | AmbO3 | *Fischerella ambigua* UTEX 1903 | AHB62761.1 | 71/81 |
| *orf2* | 336 | 2517064615 | Hypothetical protein | *Cystobacter fuscus* DSM 2262 | WP_002631743.1 | 54/71 |
| *hpiU1* | 228 | 2517064616 | AmbU1 | *Fischerella ambigua* UTEX 1903 | AHB62760.1 | 97/98 |
| *hpiU2* | 143 | 2517064617 | AmbU2 | *Fischerella ambigua* UTEX 1903 | AHB62759.1 | 100/100 |
| *hpiU3* | 219 | 2517064618 | AmbU4 | *Fischerella ambigua* UTEX 1903 | AHB62757.1 | 73/82 |
| *hpiU4* | 227 | 2517064619 | AmbU3 | *Fischerella ambigua* UTEX 1903 | AHB62758.1 | 73/82 |
| *hpiO4* | 285 | 2517064620 | AmbO4 | *Fischerella ambigua* UTEX 1903 | AHB62756.1 | 100/100 |
| *hpiO5* | 290 | 2517064621 | AmbO5 | *Fischerella ambigua* UTEX 1903 | AHB62755.1 | 97/98 |
| *hpiE1* | 388 | 2517064622 | AmbE1 | *Fischerella ambigua* UTEX 1903 | AHB62754.1 | 98/99 |
| *hpiE2* | 397 | 2517064623 | AmbE2 | *Fischerella ambigua* UTEX 1903 | AHB62753.1 | 99/100 |
| *hpiE3* | 151 | 2517064624 | AmbE3 | *Fischerella ambigua* UTEX 1903 | AHB62752.1 | 100/100 |
| *hpiO6* | 386 | 2517064625 | Alkanesulfonate monooxygenase | *Fischerella muscicola* | WP-016867816.1 | 94/97 |
| *orf2* | 277 | 2517064626 | Phosphate ABC transporter ATP-binding protein | *Fischerella muscicola* | WP_016867814.1 | 91/96 |
| *orf3* | 298 | 2517064627 | Phosphate ABC transporter permease | *Fischerella muscicola* | WP_016867813.1 | 93/96 |
| *orf4* | 331 | 2517064628 | Phosphate ABC transporter permease | *Fischerella muscicola* | WP_016867812.1 | 95/96 |
| *orf5* | 333 | 2517064629 | Phosphate-binding protein | *Fischerella muscicola* | WP_016867811.1 | 94/96 |
| *orf6* | 385 | 2517064630 | Sulfate-binding protein | *Fischerella muscicola* | WP_016867810.1 | 88/93 |
| *orf7* | 395 | 2517064630 | Hypothetical protein | *Fischerella muscicola* | WP_016867802.1 | 89/95 |
| ^*^  Size of encoded protein | | | | | | |

Additional File 1, Table S6: BLASTx analysis of the welwitindolinone (*wel*) gene cluster in *Fischerella* sp*.* PCC 9431:

| **Gene** | **Size^*^ (aa)** | **IMG Gene ID** | **Top BLASTx**  **(nucleotide-protein) hit** | **Organism** | **Accession number** | **Identity/**  **Similarity** |
| --- | --- | --- | --- | --- | --- | --- |
| *welO15* | 290 | 2512981112 | AmbO5 | *Fischerella ambigua* UTEX 1903 | AHB62755.1 | 78/88 |
| *welM1* | 347 | 2512981114 | Hypothetical protein | *Fischerella* sp*.* PCC 9339 | WP_017307678.1 | 55/72 |
| *welU8* | 226 | 2512981115 | AmbU3 | *Fischerella ambigua* UTEX 1903/ | AHB62758.1 | 61/72 |
| *welO14* | 359 | 2512981116 | AmbO1 | *Fischerella ambigua* UTEX 1903/ | AHB62763.1 | 67/80 |
| *welO^£^* | *^£^* | 2512981118  2512981117 | HpiO8 | *Fischerella* sp*.* PCC 9339 | WP_017308631.1 | 65/81 |
| *welO^£^* | *^£^* | 2512981120  2512981119 | HpiO8 | *Fischerella* sp*.* PCC 9339 | WP_017308631.1 | 72/84 |
| *welO12* | 357 | 2512981121 | HpiO8 | *Fischerella* sp*.* PCC 9339 | WP_017308631.1 | 72/83 |
| *welU6* | 228 | 2512981122 | HpiU6 | *Fischerella* sp*.* PCC 9339 | WP_017308630.1 | 93/96 |
| *welD4* | 419 | 2512981123 | HpiD4 | *Fischerella* sp*.* PCC 9339 | WP_017308629.1 | 97/99 |
| *welP2* | 334 | 2512981124 | HpiP2 | *Fischerella* sp*.* PCC 9339 | WP_017308628.1 | 89/93 |
| *welR1* | 234 | 2512981125 | AmbR1/HpiR1 | *Fischerella ambigua* UTEX 1903/ *Fischerella* sp*.* PCC 9339 | AHB62767.1 / WP_017308626.1 | 91/95 |
| *welR2* | 243 | 2512981126 | HpiR2 | *Fischerella* sp*.* PCC 9339 | WP_017308625.1 | 94/95 |
| *welR3* | 363 | 2512981127 | WelR3 | *Fischerella muscicola* | WP_016862237.1 | 87/93 |
| *welC3* | 214 | 2512981128 | HpiC3 | *Fischerella* sp*.* PCC 9339 | WP_01738609.1 | 96/98 |
| *welT5* | 365 | 2512981129 | AmbT5 | *Fischerella ambigua* UTEX 1903 | AHB62782.1 | 93/96 |
| *welT4* | 415 | 2512981130 | WelT4 | *Fischerella muscicola* | WP_016862234.1 | 96/98 |
| *welT3* | 274 | 2512981131 | WelT3 | *Fischerella muscicola* | WP_016862233.1 | 90/95 |
| *welT2* | 283 | 2512981132 | HpiT2 | *Fischerella* sp*.* PCC 9339 | WP_017308613.1 | 92/96 |
| *welT1* | 734 | 2512981133 | AmbT1 | *Fischerella ambigua* UTEX 1903 | AHB62778.1 | 91/95 |
| *welC2* | 301 | 2512981134 | AmbC2 | *Fischerella ambigua* UTEX 1903 | AHB62777.1 | 98/98 |
| *welD3* | 408 | 2512981135 | AmbD3 / HpiD3 | *Fischerella ambigua* UTEX 1903 / *Fischerella* sp*.* PCC 9339 | AHB62776.1 / WP_017308616.1 | 99/99 |
| *welD2* | 647 | 2512981136 | AmbD2 | *Fischerella ambigua* UTEX 1903 | AHB62775.1 | 98/98 |
| *welP1* | 308 | 2512981137 | AmbP1 / HpiP1 | *Fischerella ambigua* UTEX 1903 / *Fischerella* sp*.* PCC 9339 | AHB62774.1 / WP_017308618.1 | 96/97 |
| *welI3* | 273 | 2512981139 | HpiI3 | *Fischerella* sp*.* PCC 9339 | WP_017308619.1 | 97/98 |
| *welI2* | 331 | 2512981140 | AmbI2 | *Fischerella ambigua* UTEX 1903 | AHB62772.1 | 95/97 |
| *welI1* | 319 | 2512981142 | AmbI1 / HpiI1 | *Fischerella ambigua* UTEX 1903 / *Fischerella* sp*.* PCC 9339 | AHB62771.1 / WP_017308621.1 | 92/97 |
| *welS2* | 170 | 2512981143 | Transposase | *Nostoc* sp. PCC 7107 | YP_007048221.1 | 85/89 |
| *welD1* | 406 | 2512981144 | AmbD1 / HpiD1 | *Fischerella ambigua* UTEX 1903 / *Fischerella* sp*.* PCC 9339 | AHB62770.1 / WP_017308622.1 | 95/98 |
| *welC1* | 192 | 2512981145 | AmbC1 / HpiC1 | *Fischerella ambigua* UTEX 1903 / *Fischerella* sp*.* PCC 9339 | AHB62769.1 / WP_017308623.1 | 94/97 |
| *welM2* | 256 | 2512981146 | WelM2 | *Fischerella muscicola* | WP_016862218.1 | 84/92 |
| *orf1* | 359 | 2512981147 | Orf1 | *Fischerella muscicola* | WP_016862217.1 | 97/98 |
| *orf2* | 74 | 2512981148 | Hypothetical protein | *Fischerella* sp*.* PCC 9339 | WP_017308491.1 | 93/94 |
| *orf3* | 903 | 2512981149 | Hypothetical protein Npun_F2218 | *Nostoc punctiforme* PCC 73102 | YP_001865757.1 | 87/93 |
| *welH* | 542 | 2512981150 | Hypothetical protein | *Fischerella* sp*.* PCC 9339 | WP_017308202.1 | 72/85 |
| *welM3* | 279 | 2512981151 | Hypothetical protein  (N-methyltransferase) | *Fischerella* sp*.* JSC-11 | WP_009458912.1 | 94/97 |
| *welE4* | 105 | 2512981152 | Small multidrug resistance protein | *Fischerella* sp*.* JSC-11 | WP_009458415.1 | 79/90 |
| *orf4* | 136 | 2512981153 | Hypothetical protein | *Oscillatoria* sp*.* PCC 10802 | WP_017715310.1 | 45/66 |
| *orf5* | 252 | 2512981154 | Short chain dehydrogenase | *Gloeobacter violaceus* PCC 7421 | NP_925784.1 | 62/80 |
| *welO16* | 382 | 2512981155 | Hypothetical protein | *Calothrix* sp. PCC 7103 | WP_019496741.1 | 57/74 |
| *orf6* | 237 | 2512981156 | Hypothetical protein | *Calothrix* sp. PCC 7103 | WP_019496743.1 | 50/70 |
| *orf7* | 277 | 2512981157 | Hypothetical protein | *Fischerella* sp*.* PCC 9339 | WP_017307918.1 | 80/89 |
| *wel017* | 518 | 2512981158 | Hypothetical protein | *Scytonema hofmanni* | WP_017741725.1 | 89/94 |
| *orf8* | 171 | 2512981159 | DoxX protein | *Nostoc* sp. PCC 7524 | YP_007075410.1 | 90/95 |
| *orf9* | 471 | 2512981160 | NADH dehydrogenase, FAD-containing subunit | *Nostoc* sp. PCC 7524 | YP_007075409.1 | 88/94 |
| *orf11* | 628 | 2512981161 | Hypothetical protein | *Synechcoccus* sp*.* PCC 7336 | WP_017327159.1 | 33/55 |
| ^*^ Size of gene  *^£^*Gene name and size of encoded protein are unable to be determined due to N’s in nucleotide sequence. | | | | | | |

Additional File 1, Table S7: BLASTx analysis of the welwitindolinone (*wel*) gene cluster in *Fischerella muscicola* SAG 1427-1:

| **Gene** | **Size^*^ (aa)** | **IMG Gene ID** | **Top BLASTx**  **(nucleotide-protein) hit** | **Organism** | **Accession number** | **Identity/**  **Similarity** |
| --- | --- | --- | --- | --- | --- | --- |
| *orf13* | 1965 | 2550701587 | Hypothetical protein | *Chlorogloeopsis fritschii* | WP_016876159.1 | 29/47 |
| *welR3* | 369 | 2550701586 | Response regulator with CheY-like receiver domain and winged-helix DNA-binding domain | *Microcoleus* sp*.* PCC 7113 | YP_007122524.1 | 52/73 |
| *welC3* | 214 | 2550701585 | HpiC3 | *Fischerella* sp*.* PCC 9339 | WP_01738609.1 | 96/98 |
| *welT5* | 365 | 2550701584 | HpiT5 | *Fischerella* sp*.* PCC 9339 | WP_01738610.1 | 96/97 |
| *welT4* | 415 | 2550701583 | AmbT4 | *Fischerella ambigua* UTEX 1903 | AHB62781.1 | 96/97 |
| *welT3* | 274 | 2550701582 | HpiT3 | *Fischerella* sp*.* PCC 9339 | WP_017308612.1 | 94/94 |
| *welT2* | 283 | 2550701581 | HpiT2 | *Fischerella* sp*.* PCC 9339 | WP_017308613.1 | 93/96 |
| *welT1* | 734 | 2550701580 | AmbT1 | *Fischerella ambigua* UTEX 1903 | AHB62778.1 | 94/96 |
| *welC2* | 301 | 2550701579 | AmbC2 | *Fischerella ambigua* UTEX 1903 | AHB62777.1 | 98/99 |
| *welO18* | 486 | 2550701578 | Monooxygenase | *Calothrix* sp*.* PCC 7507 | YP_007067927.1 | 65/80 |
| *welO19* | 358 | 2550701577 | AmbO1 | *Fischerella ambigua* UTEX 1903 | AHB62763.1 | 73/82 |
| *welD3* | 408 | 2550701576 | AmbD3 / HpiD3 | *Fischerella ambigua* UTEX 1903 / *Fischerella* sp*.* PCC 9339 | AHB62776.1 / WP_017308616.1 | 99/99 |
| *welD2* | 648 | 2550701575 | AmbD2 | *Fischerella ambigua* UTEX 1903 | AHB62775.1 | 96/98 |
| *welP1* | 308 | 2550701574 | AmbP1 / HpiP1 | *Fischerella ambigua* UTEX 1903 / *Fischerella* sp*.* PCC 9339 | AHB62774.1 / WP_017308618.1 | 96/97 |
| *welI3* | 273 | 2550701572 | HpiI3 | *Fischerella* sp*.* PCC 9339 | WP_017308619.1 | 92/97 |
| *welI2* | 331 | 2550701571 | AmbI2 | *Fischerella ambigua* UTEX 1903 | AHB62772.1 | 96/98 |
| *welI1* | 319 | 2550701569 | AmbI1 / HpiI1 | *Fischerella ambigua* UTEX 1903 / *Fischerella* sp*.* PCC 9339 | AHB62771.1 / WP_017308621.1 | 89/96 |
| *welD1* | 285 | 2550701568 | AmbD1 / HpiD1 | *Fischerella ambigua* UTEX 1903 / *Fischerella* sp*.* PCC 9339 | AHB62770.1 / WP_017308622.1 | 94/97 |
| *welC1* | 192 | 2550701566 | AmbC1 / HpiC1 | *Fischerella ambigua* UTEX 1903 / *Fischerella* sp*.* PCC 9339 | AHB62769.1 / WP_017308623.1 | 86/92 |
| *welM2* | 254 | 2550701565 | SAM-dependent methyltransferase | *Rhodococcus* | WP_005260767 | 29/52 |
| *orf1* | 359 | 2550701564 | 6- phosphofrucktokinase | *Nostoc* sp*.* PCC 7120 | YP_227636.1 | 91/94 |
| *orf12* | 846 | 2550701562 | Acyl-CoA dehydrogenase | *Fischerella muscicola* | WP_016868810.1 | 81/90 |
| ^*^ Size of encoded protein | | | | | | |
