# Supplementary material for: Comparative analysis of hapalindole, ambiguine and welwitindolinone gene clusters and reconstitution of indole-isonitrile biosynthesis from cyanobacteria
Source: BMC Microbiol. 2014 Aug 1;14:213. doi: 10.1186/s12866-014-0213-7 (PMC4236562; doi:10.1186/s12866-014-0213-7)
Supplement: Additional file 2: — Phylogenetic analysis of HpiP1/AmbP1/WelP1 enzyme. [file s12866-014-0213-7-S2.docx]

**
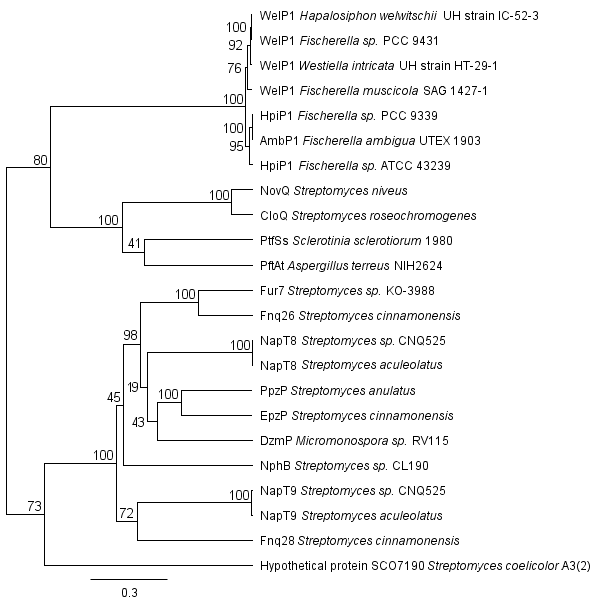
**

**Additional File 2: Phylogenetic analysis of HpiP1/AmbP1/WelP1 enzyme.** Phylogenetic tree was constructed using a 315 amino acid fragment of HpiP1/AmbP1/WelP1 from the cyanobacterial strains analyzed in this study and other members of the ABBA superfamily of prenyltransferases. NovQ from *Streptomyces niveus* [GenBank: AAF67510], CloQ from *Streptomyces roseochromogenes* [GenBank: AAN65239], PtfSs from *Sclerotinia sclerotiorum* 1980 [GenBank: EDN93598], PftAt from *Aspergillus terreus* NIH2624 [GenBank: EAU39467], Fur7 from *Streptomyces* sp*.* KO-3988 [GenBank: BAE78975], Fnq26 from *Streptomyces cinnamonensis* [GenBank: CAL34104], NapT8 from *Streptomyces* sp*.* CNQ525 [GenBank: ABS50489], NapT8 from *Streptomyces aculeolatus* [GenBank: ABS50461], PpzP from *Streptomyces anulatus* [GenBank: CAX48655], EpzP from *Streptomyces cinnamonensis* [GenBank: ADQ43372], DzmP from *Micromonospora sp.* RV115 [GenBank: AHG27152], NphB from *Streptomyces* sp*.* CL190 [GenBank: BAE00106], NapT9 from *Streptomyces* sp*.* CNQ525 [GenBank: ABS50490], NapT9 from *Streptomyces aculeolatus* [GenBank: ABS50462], Fnq28 from *Streptomyces cinnamonensis* [GenBank: CAL34106], and hypothetical protein SC07190 from *Streptomyces coelicolor* A3(2). Phylogenetic tree was constructed using the Geneious Tree Builder program, using the neighbour-joining method. Numbers at each branch point are the bootstrap values for percentages of 100 replicate trees.
