# Supplementary material for: Comparative analysis of hapalindole, ambiguine and welwitindolinone gene clusters and reconstitution of indole-isonitrile biosynthesis from cyanobacteria
Source: BMC Microbiol. 2014 Aug 1;14:213. doi: 10.1186/s12866-014-0213-7 (PMC4236562; doi:10.1186/s12866-014-0213-7)
Supplement: Additional file 3: — Sequence alignment and identification of conserved motifs from isonitrile proteins I1and I2. [file s12866-014-0213-7-S3.pdf]

```
                                                          I
PvcA                   QVQLPRIAAFIEAGEPIEFVLPAFPAKSPNPGKVLDSRPDMAERLSLSFLNHLCQRIQLF
IsnA                   APHLSKVRRAIERREPIHLVLPAFPAKSPSRRKTFGPLPDKAEELALESLQSLCNAIQLL
FS43239_HpiI1          APHLPKIQSFIENNEPIHFILPAFPAKSPNPQKVLGPMPDMGERVALQFLQNLCNQISEI
FS43239_HpiI2          APHLDKVQYFVANNEPIHFILPAFPAKSPNTQKVLGTMPDMGEQVSLKFLQSLCDQISEI
FS9339_HpiI1           APHLPKIQSFIENNEPIHFILPAFPAKSPNPQKVLGPMPDMGERVALQFLQNLCNQISEI
FS9339_HpiI2           APHLEKVQYFVEHNEPIHFILPAFPAKSPNTQKVLGTMPDMGEQVSLKFLQSLCDQISEI
FA_AmbI1               APHLPKIQSFIENNEPIHFILPAFPAKSPNPQKVLGPMPDMGERVALQFLQNLCNQISEI
FA_AmbI2               APHLEKVQYFVEHNEPIHFILPAFPAKSPNTQKVLGTMPDMGEQVSLKFLQSLCDQISEI
HW_WelI1               APHIPKIQSFIESNEPIHFILPAFPAKSPNPQKVLGPMPDMGERVALQFLQNLCNHISQI
HW_WelI2               APHLDKVQYFVENNEPIHFILPAFPAKSPNTQKVLGTMPDMGEQVSLKFLQSLCDQISEI
WI_WelI1               APHIPKIQSFIESNEPIHFILPAFPAKSPNPQKVLGPMPDMGERVALQFLQNLCNHISQI
WI_WelI2               APHLDKVQYFVENNEPIHFILPAFPAKSPNTQKVLGTMPDMGEQVSLKFLQSLCDQISEI
FS9431_WelI1           APHIPKIQSFIESNEPIHFILPAFPAKSPNPQKVLGPMPDMGERVALQFLQNLCNHISQI
FS9431_WelI2           APHLDKVQYFVENNEPIHFILPAFPAKSPNTQKVLGTMPDMGEQVSLKFLQSLCDQISEI
FMSAG1427-1_WelI1      APHIPKIQSFIEHNEPIHFILPAFPAKSPNPQKVLGPMPDMGERVALQFLQNLCNQISKV
FMSAG1427-1_WelI2      APHLEKVQYFVEHNEPIHFILPAFPAKSPNTQKVLGTMPDMGELVSLKFLQSLCNQISEI
                         :: ::   :   ***.::**********. *.:   ** .* ::*. *: **: *. .

                                      II
PvcA                   YAPGAKITVCSDGRVFGDLVRIGDAHISAYQDALRLMIEEIGATHIGVFNLEDVRAFEAQ
IsnA                   YAPGARLTLCSDGRVFSDLVGVTEEDVTRYGQEIRTMIRRLGLRSLDTFHLEDRFT----
FS43239_HpiI1          YASGAKITICSDGRVFTDLVAITDENVSLYRQGIQRLLNEINADAIDTFCLENVFT----
FS43239_HpiI2          YAPGAKLTICSDGRVFSDLVGVTDENVTLYGQIIQALLKEMKADAIDVFNLEDMYT----
FS9339_HpiI1           YASGAKITICSDGRVFTDLVAITDENVSLYRQGIQRLLNEINADAIDTFCLENVFT----
FS9339_HpiI2           YAPGAKLTICSDGRVFSDLVGVTDENVTLYGQIIQALLKEMNADAIDVFNLEDMYT----
FA_AmbI1               YASGAKITICSDGRVFTDLVAITDENVSLYRQGIQRLLNEINADAIDTFCLENVFT----
FA_AmbI2               YAPGAKLTICSDGRVFSDLVGVTDENVTLYGQIIQALLKEMKADAIDVFNLEDMYT----
HW_WelI1               YAPGAKITICSDGRVFTDLVAITDENVSLYRQGIGRLLNEINADAIDTFCLENVFT----
HW_WelI2               YAPGAKLTICSDGRVFSDLVGVTDENVTLYGQIILTLLKEMKADAIDVFNLEDMYT----
WI_WelI1               YAPGAKITICSDGRVFTDLVAITDENVSLYRQGIGRLLNEINADAIDTFCLENVFT----
WI_WelI2               YAPGAKLTICSDGRVFSDLVGVTDENVTLYGQIILTLLKEMKADAIDVFNLEDMYT----
FS9431_WelI1           YAPGAKITICSDGRVFTDLVAITDENVSLYRQGIGRLLNEINADTIDTFCLENVFT----
FS9431_WelI2           YAPGAKLTICSDGRVFSDLVGVTDENVTLYGQIILTLLKEMKADAIDVFNLEDMYT----
FMSAG1427-1_WelI1      YPPGAKITICSDGRVFTDLVAITDENVSLYRQGIERLLNEINADRIDTFCLENVFS----
FMSAG1427-1_WelI2      YAPGAKLTICSDGRVFSDLVAVTDENVTLYGQRIQALLKEMEADAINVFNLEDMYT----
                       *  **::*:******* *** : : .:: * : :  ::..:    : .* **:  :

                                                                     III
PvcA                   RDNHEQLRQLLIGGYAEPLESIRETLLASEEGLLLYRAITRFLYEDGLTPDYQ-GSKTAL
IsnA                   GDTFQEMRDLMETQYAEPYAQFAARVQKD--NPILLNGIERFLLEERSPDAPA-LSKTQA
FS43239_HpiI1          GMSFDQMRKTLVKQYAQPIESIQERVNSEDKHRQFFKGIYHLLFDDYLVLYPD-KSREQI
FS43239_HpiI2          DLSFDEMRQKLVKLYGQTIEAIKDAVKNNDHQCQMFNGIHRFLVEDYQVLEADK-SRNKI
FS9339_HpiI1           GMSFDQMRKTLVKQYAQPIESIQERVNSEDKHRQFFKGIYHLLFDDYLVLYPD-KSREQI
FS9339_HpiI2           DLSFDEMRQKLVKLYGQTIEAIKDAVKNNEHQCQMFNGIHRFLVEDYQVLEADKKSRNKI
FA_AmbI1               GMSFDQMRKTLVKQYAQPIESIQERVNSEDKHRQFFKGIYHLLFDDYLVLYPD-KSREQI
FA_AmbI2               DLSFDEMRQKLVKLYGQTIEAIKDAVKNNDHQCQMFNGIHRFLVEDYQVLEAHK-SRNKI
HW_WelI1               GMSFDQMRKTLVEQYAQPIESIQERVNSEDKHRQFFNGIYHLLFDDYLVLYPD-KSREQI
HW_WelI2               DLSFDQMRHKLVKLYGQSIEAIKDAVKNNEHQCQMFNGIHRFLVEDYQVLEAH-KSRNKI
WI_WelI1               GMSFDQMRKTLVEQYAQPIESIQERVNSEDKHRQFFNGIYHLLFDDYLVLYPD-KSREQI
WI_WelI2               DLSFDQMRHKLVKLYGQSIEAIKDAVKNNEHQCQMFNGIHRFLVEDYQVLEAH-KSRNKI
FS9431_WelI1           GMSFDQMRKTLVEQYAQPIESIQERVNSEDKHRQFFNGIYHLLFDDYLVLYPD-KSREQI
FS9431_WelI2           DLSFDQMRHKLVKLYGQSIEAIKDAVKNNEHQCQMFNGIHRFLVEDYQVLEAH-KSRNKI
FMSAG1427-1_WelI1      RMSFDEMRKTLIKQYSQPIESIQERVNSEDKHRQFFNGIYHLLFDDYLVLYPD-KPRNQV
FMSAG1427-1_WelI2      DLSFDEMRQKLVKLYGQNIEAIKEAVKNDDHHCQMFNGIHRFLVEDYQVLEAH-KSRNKI
                         ..:::*. :   *.:    :   :  .     : ..* ::* ::          :

                                    IV               V                         VI
PvcA                   QRDAKERAYGVIQRSWAWGALLADQFPRAIRLSIHPQPADSLKFGIHMMP--TRDWLTPWHG
IsnA                   RKEARTRAIEVVRRSEAWGRLIAERFPSALRLSIHPQMPHSPKIGLRMGEQGKDLWITPWHG
FS43239_HpiI1          EVECNLRAYEVIQRSNAWTTLVGQHFPQSLRLSIHPQDYHSNKIGIHMIK-TSDQWCTPWHN
FS43239_HpiI2          RLECKARAYEVIQRSNAWSVLISELYPHSVRLSIHPQHYHSEKIGIHMIK-TLDQWCTPWHN
FS9339_HpiI1           EVECNLRAYEVIQRSNAWTTLVGQHFPQSLRLSIHPQDYHSNKIGIHMIK-TSDQWCTPWHN
FS9339_HpiI2           RLECKTRAYEVIQRSNAWSVLISELYPHSVRLSIHPQHYHSEKIGIHMIK-TLDQWCTPWHN
FA_AmbI1               EVECNLRAYEVIQRSNAWTTLVGQHFPQSLRLSIHPQDYHSNKIGIHMIK-TSDQWCTPWHN
FA_AmbI2               RLECKTRAYEVIQRSNAWSVLISELYPHSVRLSIHPQHYHSEKIGIHMIK-TLDQWCTPWHN
HW_WelI1               EIECNVRAYEVIQRSNAWTTLVGQHFPQSLRLSIHPQDYHSNKIGIHMIK-TSDQWCTPWHN
HW_WelI2               RLECKARAYEVIQRSNAWSVLISELYPHSVRLSIHPQHYHSEKIGIHMIK-TLDQWCTPWHN
WI_WelI1               EIECNVRAYEVIQRSNAWTTLVGQHFPQSLRLSIHPQDYHSNKIGIHMIK-TSDQWCTPWHN
WI_WelI2               RLECKARAYEVIQRSNAWSVLISELYPHSVRLSIHPQHYHSEKIGIHMIK-TLDQWCTPWHN
FS9431_WelI1           EIECNVRAYEVIQRSNAWTTLVGQHFPQSLRLSIHPQDYHSNKIGIHMIK-TSDQWCTPWHN
FS9431_WelI2           RLECKARAYEVIQRSNAWSVLISELYPHSVRLSIHPQHYHSEKIGIHMIK-TLDQWCTPWHN
FMSAG1427-1_WelI1      EVECNVRAYEVIQRSNAWTKLVGQHFPQSLRLSIHPQHYHSDKIGIHMIK-TSDQWCTPWHN
FMSAG1427-1_WelI2      RLECKTRAYEVIQRSNAWSVLISELYPHSVRLSIHPQHYHSEKIGIHMIK-TLDQWCTPWHN
                       . :.. **  *: ** **  *:.: :* ::*******  .* *:*::*       * ****
```

Additional File 3: Sequence alignment and identification of conserved motifs from isonitrile proteins I1 and I2. All identified isonitrile proteins in *hpi*, *amb* and *wel* biosynthetic gene clusters were aligned with PvcA from *Pseudominas aeruginosa* PA01 [GenBank: AAC21671] and IsnA from uncultured organism [GenBank: AAZ39275]. The six previously identified conserved motifs are identified within the boxes [1].

1. Drake EJ, Gulick AM: **Three-dimensional structures of *Pseudomonas aeruginosa* PvcA and PvcB, two proteins involved in the synthesis of 2-isocyano-6,7-dihydroxycoumarin**. *J Mol Biol* 2008, **384**(1):193-205.
