# Supplementary material for: Comparative analysis of hapalindole, ambiguine and welwitindolinone gene clusters and reconstitution of indole-isonitrile biosynthesis from cyanobacteria
Source: BMC Microbiol. 2014 Aug 1;14:213. doi: 10.1186/s12866-014-0213-7 (PMC4236562; doi:10.1186/s12866-014-0213-7)
Supplement: Additional file 4: — Sequence alignment of isonitrile protein I3 with IsnB and PvcB. [file s12866-014-0213-7-S4.pdf]

```
FS_ATCC43239_HpiI3      MMVSTS---V-EQSTQFSVKSLTPFGALLEATEDHSDIQQLSIEQLCQLTWEHRLIVLRG
FS_PCC9339_HpiI3        MMVSTS---V-EQSAQFSVKSLTPFGALLEATEDHSDIQQLSIEQLCQLTWEHRLIVLRG
FA_UTEX1903_AmbI3       MIVSTS---V-EQSAQFSVKSLTPFGALLEATEDHSDIQQLSIEQLCQLTWEHRLIVLRG
HW_IC-52-3_WelI3        MMVSTS---V-EQSTQFSVKSLTPFGALLEANEDHSDIQQLSIEQLCQLTWEHRLIVLRG
WI_HT-29-1_WelI3        MMVSTS---V-EQSTQFSVKSLIPFGALLEANEDCSDIQQLSIEQLCQLTWEHRLIVLRG
FS_PCC9431_WelI3        MMVSTS---V-EQSTQFSVKSLTPFGALLEANEDHSDIQQLSIEQLCQLTWEHRLIVLRG
FM_SAG1427-1_WelI3      MMVSTS---L-GQSTQFSVKPLTPFGALLEATEYHSDIKQLSIEQLCELTWEHRLIVLRG
IsnB                    -MTHATLQSN-TTTERWQQQLLPTFGMLVHAREAGTPLSSLPADTLRAWAEAESLVILRG
PvcB                    MNAYLSDQPVRLSPLRDEQGNQPRFGLLLEPGRPGMHVGELPAQWLKGLARSHHLLLLRG
                          .  :         : .      ** *:.  .     : .*  : *    :  . *::***

                                                                              H D
FS_ATCC43239_HpiI3      FSLL-EREELSIYCQRWGELLVWNFGTVLDLIVHQNPENYLFTNGNVPFHWDGAFAEAVP
FS_PCC9339_HpiI3        FSLL-EREELSTYCQRWGELLVWNFGTVLDLIVHQNPENYLFTNGNVPFHWDGAFAEAVP
FA_UTEX1903_AmbI3       FSLL-EREELSTYCQRWGELLVWNFGTVLDLIVHQNPENYLFTNGNVPFHWDGAFAEAVP
HW_IC-52-3_WelI3        FSLL-EREELSTYCQRWGELLVWNFGTVLDLIVHQNPENYLFTNGNVPFHWDGAFAEAVP
WI_HT-29-1_WelI3        FSLL-EREELSTYCQRWGELLVWNFGTVLDLIVHQNPENYLFTNGNVPFHWDGAFAEAVP
FS_PCC9431_WelI3        FSLL-EREELSTYCQRWGELLVWNFGTVLDLIVHQNPENYLFTNGNVPFHWDGAFAEAVP
FM_SAG1427-1_WelI3      FSLL-EREELSTYCQRWGELLIWNFGTVLDLIVHESPENYLFTNGNVPFHWDGAFAEAVP
IsnB                    FAPP-EGDALPSYCRGLGDLLEWDFGAINNLQAQSEAKNYLFTNRAVPFHWDGAFVGRIP
PvcB                    FAAFADAESLTRYCHDFGEVMLWPFGAVLELVEQEGAEDHIFANNYVPLHWDGMYLETVP
                        *:   : : *  **:  *::: * **:: :*  :.  ::::*:*  **:* ** :   :*

FS_ATCC43239_HpiI3      RFLFFQCLKAPEAGSGGESLFCDTVRTLQNVSPQQREIWQKTEISYKTEKVAHYGGEITK
FS_PCC9339_HpiI3        RFLFFQCLKAPEAGSGGESLFCDTVRILQNVSPQQREIWQKTEISYKTQKVAHYGGEITK
FA_UTEX1903_AmbI3       RFLFFQCLKAPEAGSGGESLFCDTVRILQNVSPQQREIWQKTEISYKTQKVAHYGGEITK
HW_IC-52-3_WelI3        RFLFFQCLKAPEAGSGGESLFCDTVRILQNVSPEQREIWQKTEINYKTEKVAHYGGEITK
WI_HT-29-1_WelI3        RFLFFQCLKAPEAGSGGESLFCDTVRILQNLSPQQREIWQKTEINYKSEKVAHYGGEITK
FS_PCC9431_WelI3        RFLFFQCLKAPEAGSGGESLFCDTVRILQNVSPEQREIWQKTEINYKTEKVAHYGGEITK
FM_SAG1427-1_WelI3      RFLFFQCLKAPEAGSGGESLFCDTVRILQNLSPQQREIWEKTEVSYKTEKVAHYGGEIIK
IsnB                    HWIFFHCASAPEENTGGETLFCHTPLLLEAVSAAGRAQWENISIRYSTEKLAHYGGSFTS
PvcB                    EFQVFHCVDAPGDTDGGRTTFSSTPAALQLADSSELELWRRASARYQR-SAAHYSSRSAA
                        .: .*:* .**    **.: *. *    *:  .       *.. .  *.  . ***..

FS_ATCC43239_HpiI3      SLVIKHPITGLSTLRFAEPLND-ASVHLNPLYVEVCNLPTEEQNSFINELIENLYLPQNC
FS_PCC9339_HpiI3        SLVIKHPITGLSTLRFAEPLND-ASVHLNPLYVEVCNLPAEEQNPFINELIENLYLPQNC
FA_UTEX1903_AmbI3       SLVIKHPITGLSTLRFAEPLND-ASVHLNPLYVEVCNLPAEEQNPFINELIENLYLPQNC
HW_IC-52-3_WelI3        SLVTKHPITGLSTLRFAEPLND-ASVHLNPLYVEVCNLPTEEQNPFLNELIENLYLPQNC
WI_HT-29-1_WelI3        SLVTKHPITGLSTLRFAEPLND-ASVHLNPLYVEVCNLPTEEQNPFLNELIENLYLPQNC
FS_PCC9431_WelI3        SLVTKHPITGLSTLRFAEPLND-ASVHLNPLYVEVCNLPTEEQNPFLNELIENLYLPQNC
FM_SAG1427-1_WelI3      SLVSKHPITGLSTLRFAEPLND-ASVHLNPLYVEVCNLPAEEQNQFLSELIENLYLPQNC
IsnB                    PLLAAHPIHGQTILRYAEPVND-----LNPVHLEIQGLPEESHTAFLEGMHTRLYDPAVC
PvcB                    PIVERHPRREFPILRFCEPPVEGDASFINPSEFHYDGIAPEQRGELLASLRRCLYHPQAH
                         ::  **       **:.**  :      :**  ..    :  *.:  ::  :    ** *

                                          H                  R
FS_ATCC43239_HpiI3      FAHEWQEGDFLIADNHALLHGRNPFLSNSQRHLQRVHIL-------------
FS_PCC9339_HpiI3        FAHEWQEGDFLIADNHALLHGRNPFLSNSQRHLQRVHIL-------------
FA_UTEX1903_AmbI3       FAHEWQEGDFLIADNHALLHGRNPFLSNSQRHLQRVHIL-------------
HW_IC-52-3_WelI3        FAHEWQEGDFLIADNHALLHGRNPFLFNSQRHLQRVHIL-------------
WI_HT-29-1_WelI3        FAHEWQEGDFLIADNHALLHGRNPFLFNSQRHLQRVHIL-------------
FS_PCC9431_WelI3        FAHEWQEGDFLIADNHALLHGRNPFLFNSQRHLQRVHIL-------------
FM_SAG1427-1_WelI3      FGHEWQEGDFLVADNHALLHGRNPFLSNSQRHLQRVHIL-------------
IsnB                    YAHAWQTGDIVIADNFTLLHGRRAFLRPESRHLRRVNIL-------------
PvcB                    YAHRWRSDDLVIADNLTLLHGREAFAHRAPRHLRRVHIHAEPALRNPHLQRD
                        :.* *:  *:::*** :*****. *     ***:**.*
```

Additional File 4: Sequence alignment of isonitrile protein I3 with IsnB and PvcB. All identified proteins in *hpi, amb* and *wel* biosynthetic gene clusters were aligned with PvcB from *Pseudominas aeruginosa* PA01 [GenBank: AAC21672] and IsnB from uncultured organism [GenBank: AAZ39276]. The four previously identified amino acid residues of the active site are identified within the boxes [1].

1. Drake EJ, Gulick AM: **Three-dimensional structures of *Pseudomonas aeruginosa* PvcA and PvcB, two proteins involved in the synthesis of 2-isocyano-6,7-dihydroxycoumarin**. *J Mol Biol* 2008, **384**(1):193-205.
