# Supplementary material for: Comparative analysis of hapalindole, ambiguine and welwitindolinone gene clusters and reconstitution of indole-isonitrile biosynthesis from cyanobacteria
Source: BMC Microbiol. 2014 Aug 1;14:213. doi: 10.1186/s12866-014-0213-7 (PMC4236562; doi:10.1186/s12866-014-0213-7)
Supplement: Additional file 5: — 1 H and 13 C NMR and HRMS spectra for chemically synthesized cis and trans indole-isonitriles. [file s12866-014-0213-7-S5.docx]

**B**

**A**

**C**

**D**

**E**


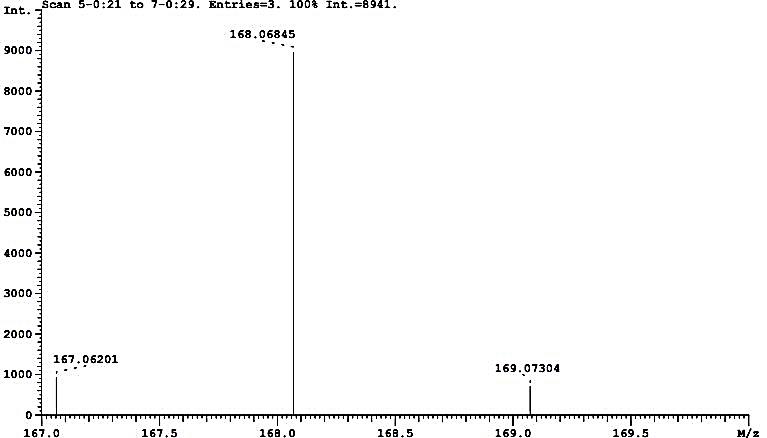


**HRMS**

Expected: 168.0687

**Additional File 5:** ^1^H and ^13^C NMR and HRMS spectra of chemically synthesized *cis* and *trans* indole-isonitrile standards. **A)** ^1^H NMR for *cis* indole-isonitrile*.* **B)** ^13^C NMR for *cis* indole-isonitrile*.* **C)** ^1^H NMR for *trans* indole-isonitrile*.* **D)** ^13^C NMR for *trans* indole-isonitrile*.* **E)** HRMS for a sample containing a mixture of *cis* and *trans* isomers.
