# Supplementary material for: Comparative analysis of hapalindole, ambiguine and welwitindolinone gene clusters and reconstitution of indole-isonitrile biosynthesis from cyanobacteria
Source: BMC Microbiol. 2014 Aug 1;14:213. doi: 10.1186/s12866-014-0213-7 (PMC4236562; doi:10.1186/s12866-014-0213-7)
Supplement: Additional file 6: — LC-ESI-MS spectrum for enzyme-catalyzed indole-isonitrile biosynthesis product. [file s12866-014-0213-7-S6.docx]

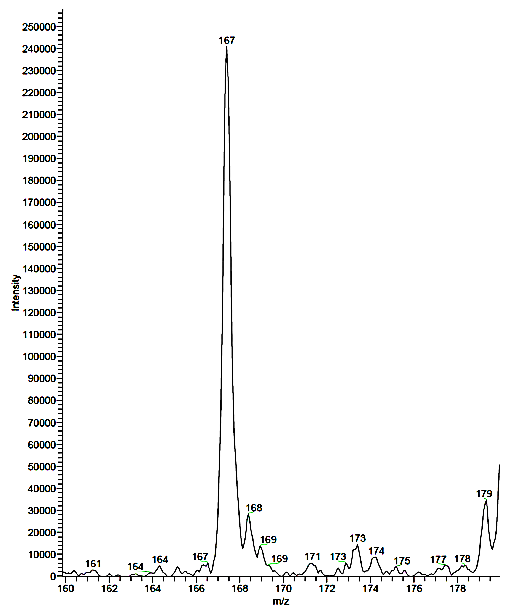


**Additional File 6:** LC-ESI-MS (negative ion mode) specta for enzyme-catalyzed indole-isonitrile biosynthesis product. Observed peak is at 167 amu. Expected peak is 167 amu for the anion shown in figure.
