# Supplementary material for: Comparative analysis of hapalindole, ambiguine and welwitindolinone gene clusters and reconstitution of indole-isonitrile biosynthesis from cyanobacteria
Source: BMC Microbiol. 2014 Aug 1;14:213. doi: 10.1186/s12866-014-0213-7 (PMC4236562; doi:10.1186/s12866-014-0213-7)
Supplement: Additional file 7: — HRESI-MS and MS peaks from LC-MS spectra for chemically synthesized indole-isonitrile and cyanobacterial extracts from FS ATCC43239 and FA UTEX1903. [file s12866-014-0213-7-S7.docx]

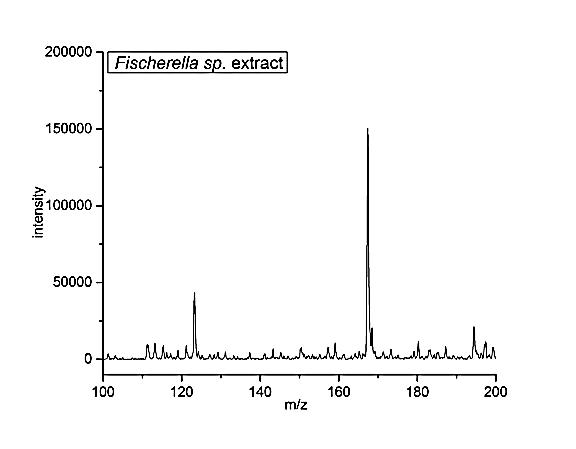

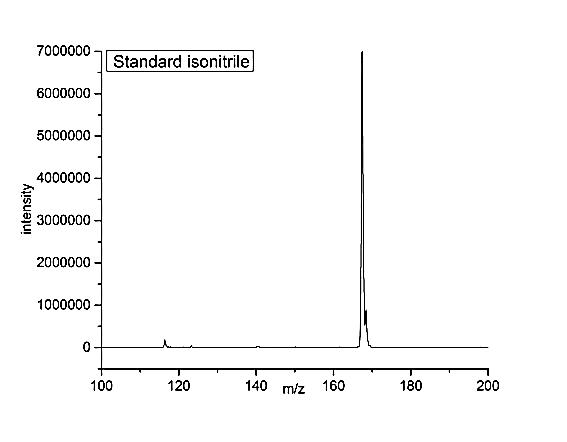

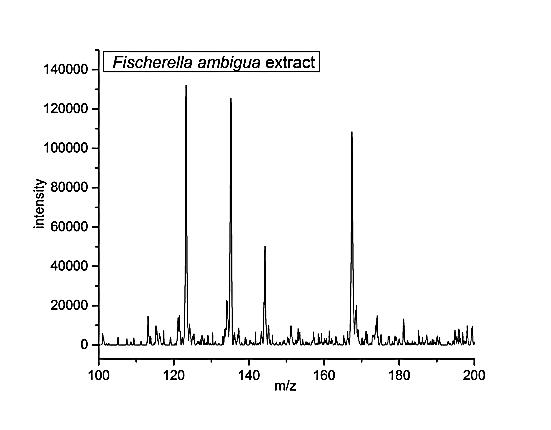

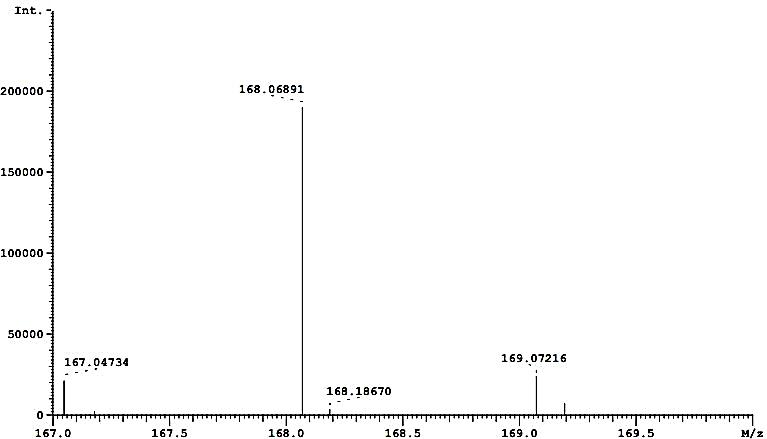

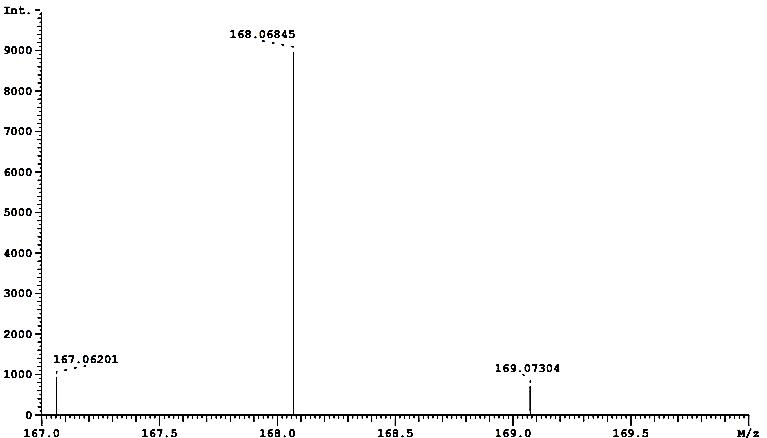


**A**

**D**

**E**

**B**

**C**

**Additional File 7**: LC-MS and HRMS data for indole-isonitrile extracted from cyanobacterial biomass of *Fischerella* cultures. A) Chemically synthesized mixture containing *cis* and *trans* isomers are seen on the MS peak at m/z = 168 amu. B) Extracts fractionated from *Fischerella* sp*.* ATCC 43239 display identical (to A) MS peak at m/z = 168 amu. C) Extracts fractionated from *Fischerella ambigua* UTEX 1903 display identical (to A) MS peak at m/z = 168 amu. D) HRMS peak with M^+^ = 168.0689 for synthesized indole-isonitrile (mixture of *cis* and *trans* isomers). E) HRMS peak at M^+^ = 168.0685 for extracted indole-isonitrile from *Fischerella* sp*.* ATCC 43239.
