# Supplementary material for: Comparative analysis of hapalindole, ambiguine and welwitindolinone gene clusters and reconstitution of indole-isonitrile biosynthesis from cyanobacteria
Source: BMC Microbiol. 2014 Aug 1;14:213. doi: 10.1186/s12866-014-0213-7 (PMC4236562; doi:10.1186/s12866-014-0213-7)
Supplement: Additional file 9: — Sequence alignment and identification of motifs from Reiske-type oxygenases. [file s12866-014-0213-7-S9.pdf]

```
                                                  #  #  #
AmbO1                   AGKPVIMERFCSHLGASLAIGEVVDGCIRCPFHHWRYDSSGVCVDIPKVAVPTTDVIPPT
AmbO2                   AGKPIIMERFCSHLGASLAIGEVVDGCIRCPFHHWRYDSSGVCVDIPKVATPTTDVIPPT
AmbO3                   AGKPVIMERFCSHLGASLAIGEVVDGCIRCPFHHWRYDSSGVCVDIPKVAAPTTDVIPPT
AmbO4                   IGKPVIMDDFS-------------------------------------------------
FS9339_HpiO4            IGKPVIMDDFS-------------------------------------------------
FS9339_HpiO8            AGKAVIMDRFCSHLGASLAIGEVVDGCIQCPFHHWRYDSSGVCVDIPKVAAPVTDQIPAT
FS43239_HpiO8           AGKAVIMDRFCSHLGASLAIGEVVDGCIQCPFHHWRYDSSGVCVDIPKVAAPVTDQIPAT
FS43239_HpiO9           AGKAVIMERFCSHLGASLAIGQLVDGCIQCPFHHWRYDNSGVCVHIPKVATPVTDQIPAT
HW_WelO11               DRKAVIMERFCSHVGASLAIGEVVDGCIQCPFHHWRYDSSGVCVHIPKVATPHTDHIPST
HW_WelO12               DRKAVIMERFCSHVGASLAIGEVVDGCIQCPFHHWRYDSSGVCVHIPKVATPHTDHIPST
HW_WelO13               DRKAVIMERFCSHVGASLAIGEVVDGCIQCPFHHWRYDHSGVCVHIPKVATPHTDHIPST
HW_WelO14               DRKAVIMERFCSHVGASLAIGEVVDGCIQCPFHHWRYDSSGVCVQIPKVATPHTDHIPST
WI_WelO11               DRKAVIMERFCSHVGASLAIGEVVDGCIQCPFHHWRYDSSGVCVHIPKVATPHTDHIPST
WI_WelO12               DRKAVIMERFCSHVGASLAIGEVVDGCIQCPFHHWRYDSSGVCVHIPKVATPHTDHIPST
WI_WelO13               DRKAVIMERFCSHVGASLAIGEVVDGCIQCPFHHWRYDSSGVCVHIPKVATPHTDHIPST
WI_WelO14               DRKAVIMERFCSHVGASLAIGEVVDGCIQCPFHHWRYDSSGVCVHIPKVATPHTDHIPST
WI_WelO19               LGKPVIMERFCSHMGASLAIGQIIDGCIQCPFHHWRYDSSGVCVDIPKVATPTTDVIPST
FS9431_WelO12           DRKAVIMERFCSHVGASLAIGEVVDGCIQCPFHHWRYDSSGVCVHIPKVATPHTDHIPST
FS9431_WelO14           DRKAVIMERFCSHVGASLAIGEVVDGCIQCPFHHWRYDSSGVCVHIPKVATPHTDHIPST
FMSAG1427-1_WelO19      LGKPVIMERFCSHMGASLAIGQIIDGCIQCPFHHWRYDSSGVCVDIPKVATPTTDVIPST

AmbO1                   ARQHTYVTQEKYGYIWVWYGSATPMFPLPKFDAAESD-KHHKYMACPASIPVKTTVRRVAE
AmbO2                   ARQHTYVTQEKYGYIWVWYGSATPMFPLPKFDAAESD-KHHKYIPYRFSLPVKTTVRRIIE
AmbO3                   ARQYTYVTQEKYGYIWVWYGTATPLFELPKFDAAEND-KHHKYMLYRFSFPVKTTVRRAIE
AmbO4                   ----TYVTREEYGYIWVWYGSVNPLFDLPVFDVTEN-NKHNYIHNYFSFTAKTSVQKILE
FS9339_HpiO4            ----TYVTREEYGYIWVWYGSVNPLFDLPVFDVTEN-NKHNYIHNYFSFTAKTSVQKILE
FS9339_HpiO8            ARQQTYVTQEKYGYIWVWYGTAHPLFDLPKFDAAESSNKHKYMSYRFFLKANTTVRRVIE
FS43239_HpiO8           ARQQTYVTQEKYGYIWVWYGTAHPLFDLPKFDAAESSNKHKYMSYRFFLKANTTVRRVIE
FS43239_HpiO9           ARQQTYVTQEKYGYIWVWYGTAHPLFDLPKFDVAESDNKHKYMPYRFSFTVKTSARRVME
HW_WelO11               ARQKTYVTEERYGYIWVWYGTATPLFELPGFDAAESNNKHKYMPYRFSFLVKTSVRRVVE
HW_WelO12               ARQKTYVTEERYGYIWVWYGTATPLFELPGFDAAES-NKHKYMPYRFSFQTETSVRRAIE
HW_WelO13               ARQKTYVTEERYGYIWVWYGTATPLFDLPKFDAAES-NKHKYMPYRFSIMVQTNVRRIIE
HW_WelO14               ARQKTYVTEERYGYIWVWYGTATPLFDLPKFDAAES-NKHRYIPYRSSFTANTSVRRVIE
WI_WelO11               ARQKTYVTEERYGYIWVWYGTATPLFELPGFDAAES-NKHKYMPYRFSFLVKTSVRRVVE
WI_WelO12               ARQKTYVTEERYGYIWVWYGTATPLFELPGFDAAES-NKHKYMPYRFSFQTETSVRRAIE
WI_WelO13               ARQKTYVTEERYGYIWVWYGTATPLFDLPKFDAAES-NKHKYMPYRFSIMVQTNVRRIIE
WI_WelO14               ARQKTYVTEERYGYIWVWYGTATPLFDLPKFDAAES-NKHRYIPYRSSFTANTSVRRVIE
WI_WelO19               AHQHTYVTQEKYGYIWVWYGSVNPLFPLPKFDPAESD-KHNYMSYRFFSKTKTTVLRVLE
FS9431_WelO12           ARQKTYVTEERYGYIWVWYGTATPLFELPGFDAAES-NKHKYMPYRFSFQTETSVRRAIE
FS9431_WelO14           ARQKTYVTEERYGYIWVWYGTATPLFDLPKFDAAES-NKHRYIPYRSSFTANTSVRRVIE
FMSAG1427-1_WelO19      ARQHTYVTQEKYGYIWVWYGSVNPLFPLPKFDPAESD-KHNYMSYRFFSKTKTTVLRVLE

                           !  !    !
AmbO1                   NAFDHHHIVSIHKMPVDGQIKLTLLNEEDVELSELPIAKEAWMGSLIEAQLKTYFGVNI-
AmbO2                   NAFDHHHLVTSHKMPIDGQIKLTLLNEDNVELSELPIAKEAWIGDIMEGQLKTYLGTRI-
AmbO3                   NAFDHHHLITMHNLPVLDQIRLTMLNEEDVELSELPFAKEAWMGSFIEARIKSFIGVGA-
AmbO4                   IVLNHHHLIRSHNSLVIDRIEHTYLDEKNVELSKLPITKEAWFGTITETQIKDYFGINE-
FS9339_HpiO4            IVLNHHHLIRSHNSLVIDRIEHTYLDEKNVELSKLPITKEAWFGTITETQIKDYFGINE-
FS9339_HpiO8            NAFDHHHLVTIHHIDVADQIELTLLNKEDIELGEIPVIKEAWIGSILKARIKNNVGVGA-
FS43239_HpiO8           NAFDHHHLVTIHHIDVADQIELTLLNKEDIELGEIPVIKEAWIGSILKARIKNNVGVGA-
FS43239_HpiO9           NVFDHQHLVTLHGMAVNGQIGLTLLNERDVEPDKLSISKEAWFGCTMEAEIKSYMSAGS-
HW_WelO11               NAFDHHHFVTVHNVPVADSIDLTLVNQKDTELSELPIAKEAWFGTVIEARIKTLTGVGAA
HW_WelO12               NGFDHHHFVSVHGLPVIDQIEMTLLDEKDAEFSELILAKEAWIGSKLDARIKNFIGVGA-
HW_WelO13               NSCDHHHLVTIHDMQVNDSVKLTVLDEKDVELSELPIAKEAWMGFIVEARIKTFLGVRG-
HW_WelO14               NIFDHHHLVAVHDMQVNDLIDLTLLDEKDIELSELPIAKEAWFGCKIEANMKAFLGVRG-
WI_WelO11               NAFDHHHFVTVHNVPVADSIDLTLVNHKDTELSELPIAKEAWFGTVIEARIKTLTGVGAA
WI_WelO12               NGFDHHHFVSVHGLPVIDQIEMTLLDEKDAEFSELILAKEAWIGSKLDARIKNFIGVGA-
WI_WelO13               NSCDHHHLVTIHDMQVNDSVKLTVLDEKDVELNKLPIAKEAWMGFIVEARIKTFLGVRG-
WI_WelO14               NIFDHHHLVAVHDMQVNDLINLTLLDEKDVELSELPITKEAWFGCKIEANMKAFLGVRG-
WI_WelO19               NSFDHHHVVATHNLPVINQIKQTLLNKEDIKISELSIAKEAWFGTVMEAQIKSYAGVGA-
FS9431_WelO12           NGFDHHHFVSVHGLPVIDQIEMTLLDEKDAEFSELILAKEAWIGSKLDARIKNFIGVGA-
FS9431_WelO14           NIFDHHHLVAVHDMQVNDLIDLTLLDEKDIELSELPIPKEAWFGCKIEANMKAFLGVRG-
FMSAG1427-1_WelO19      NSFDHHHVVVTHNLPIINQIKQTLLNKEDIKISELSIAKEAWFGTVMEAQIKSYAGVGA-
```

Additional File 9: Sequence alignment and identification of motifs from Reiske-type oxygenases. All identified Reiske-type oxygenase proteins from the *hpi*, *amb* and *wel* gene clusters were aligned. The [2Fe-2S] cluster motif is identified within the box. The iron-sulfur Reiske domain (CXXC and CXXH) is marked with #, and the mononuclear nonheme Fe(II)-binding motif (DXXHXXXXXH) is marked with !. These motifs are typical of Reiske oxygenases. The absence of the iron-sulfur cluster motif suggests AmbO4 and HpiO4 are atypical Reiske-homologous proteins.
