# Supplementary material for: Comparative analysis of hapalindole, ambiguine and welwitindolinone gene clusters and reconstitution of indole-isonitrile biosynthesis from cyanobacteria
Source: BMC Microbiol. 2014 Aug 1;14:213. doi: 10.1186/s12866-014-0213-7 (PMC4236562; doi:10.1186/s12866-014-0213-7)
Supplement: Additional file 11: — Specific primers used in this study. [file s12866-014-0213-7-S11.docx]

**Additional File 11:** Specific primers used in this study.

| **Primer** | **Sequence** | **T_m_ ( °C)** | **Reference** |
| --- | --- | --- | --- |
| HWwelO12F | GTTTTTAAGTGGCACGATGTTAC | 61.2 | This study |
| HWwelO12R | CAAAGAACCAGAAACTCAAATTAAG | 60.8 | This study |
| HWwelO12F2 | GCCTAATTCTTTGGATTCC | 57.6 | This study |
| HWwelO12R2 | CTTTACCAGGATTGATGAATG | 58.5 | This study |
| HWwelO12F3 | CTATTGGTTTTTTGTCTAGTTC | 53.9 | This study |
| HWwelO12R3 | GAACTAGACAAAAAACCAATAG | 53.9 | This study |
| HWwelO12F4 | GATTGTACTTAAAACTCCACTAGG | 56.7 | This study |
| HWwelO12R4 | CAAGATTGCAAGTTTTAATTATG | 57.1 | This study |
| HWwelO12R5 | CAATCTGGATGTTCTGGTCTTG | 63.5 | This study |
| HWwelO11R6 | CTTATGGCTAAATCTACTTTC | 51.8 | This study |
| FShpiT2F1 | CAAGTTCAGCCAGAGTATG | 55.8 | This study |
| FShpiT2F2 | GCAATTAAGCTCGGTTTAC | 56.5 | This study |
| FShpiT2F3 | GCAGGTTTATGAAGTACAG | 52.1 | This study |
| FShpiT2F4 | CCAGAGTTGAGGAAGTAAAC | 55.4 | This study |
| FShpiT1R | CTTTATCAAATCTCAAGCTGAAC | 58.6 | This study |
| FShpiT1R2 | CGTAATTATGGGTATCGAAC | 56.0 | This study |
| FShpiT1R3 | CAAAACATACAGCAGATTG | 54.0 | This study |
| FShpiT1R4 | CTACAAAAACAAGGTGGAG | 54.7 | This study |
| WIWel013F | GCTTATTGCTTTCTGCGG | 61.1 | This study |
| WIWelM1F | TCAACCGATCCTTACAC | 53.8 | This study |
| WIWelO13R | CAGCGAGTTGGTATATTGC | 58.3 | This study |
| ssuEF | GGAGAGCATATGCGTGTCATCACC | 70.6 | [[1](#_ENREF_1)] |
| ssuER | GTAAAGCTTTTACGCATGGGCATT | 67.4 | [[1](#_ENREF_1)] |
| 27F | GCTTCGGCACGGCTCGGGTCGA | 80.1 | [[2](#_ENREF_2)] |
| 809R | AGAGTTTGATCCTGGCTCAG | 61.0 | [[2](#_ENREF_2)] |
| 740F | GGCYRWAWCTGACACTSAGGGA | 55.8 | [[3](#_ENREF_3)] |
| 1494R | TACGGCTACCTTGTTACGAC | 59.3 | [[3](#_ENREF_3)] |
| WI/HW_898R | CCTTTGAGTTTCACACTTG | 55.7 | This study |
| F16srDNAF | CTCTGTGTGCCTAGGTATCC | 53.3 | This study |
| F16srDNAR | GGGGAATTTTCCGCAATGGG | 66.1 | This study |

1. Dorrestein PC, Yeh E, Garneau-Tsodikova S, Kelleher NL, Walsh CT: **Dichlorination of a pyrrolyl-S-carrier protein by FADH2-dependent halogenase PltA during pyoluteorin biosynthesis**. *Proc Natl Acad Sci USA* 2005, **102**(39):13843-13848.

2. Moffitt MC, Neilan BA: **On the presence of peptide synthetase and polyketide synthase genes in the cyanobacterial genus Nodularia**. *FEMS Microbiol Lett* 2001, **196**(2):207-214.

3. Neilan BA, Jacobs D, Therese DD, Blackall LL, Hawkins PR, Cox PT, Goodman AE: **rRNA Sequences and evolutionary relationships among toxic and nontoxic cyanobacteria of the genus *Microcystis***. *Int J Syst Bacteriol* 1997, **47**(3):693-697.
